# Supplementary material for: The Evolution and Characterization of the RNA Interference Pathways in Lophotrochozoa
Source: Genome Biol Evol. 2024 May 7;16(5):evae098. doi: 10.1093/gbe/evae098 (PMC11114477; doi:10.1093/gbe/evae098)
Supplement: evae098_Supplementary_Data [file evae098_supplementary_data.zip › Supplementary_materials.zip!/selected_miRNAs/NoGe_selected_miRNAs/mirdeep_pdf_files/NMRB01000131.1_Notospermus_geniculatus_strain_Ushimado_scaffold131,_whole_genome_shotgun_sequence_5275.pdf]

Provisional ID : NMRB01000131.1_Notospermus_geniculatus_strain_Ushimado_scaffold131,_whole_genome_shotgun_sequence_5275

Score total	:	221374.3
Score for star read(s)	:	3.9
Score for read counts	:	221365.7
Score for mfe	:	1.7
Score for randfold	:	0
Score for cons. seed	:	3
Total read count	:	434211
Mature read count	:	433367
Loop read count	:	0
Star read count	:	844

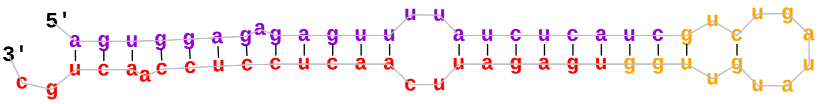

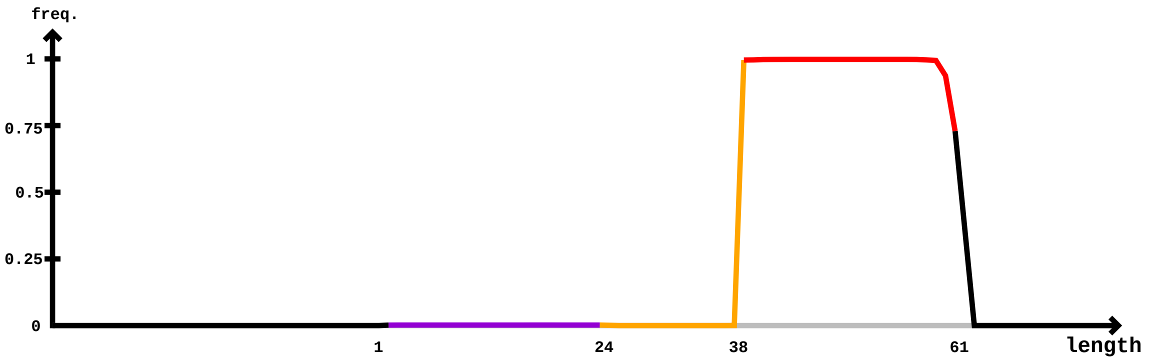

Star Mature

```
5'- ggcuccuucccuucuuacugacacaucaagagaaguggagagaguuuuaucucaucgucugauauguuggugagauucaacuccuccaacugcucucggugugaaccau   -3'   obs
    ggcuccuucccuucuuacugacacaucaagagaaguggagagaguuuuaucucaucgucugauauguuggugagauucaacuccuccaacugcucucggugugaaccau         exp
    .....................((((((.((((.(((((((.(((((..(((((((((.(......).)))))))))..))))))))).))).)))).))))))......   reads   mm   sample
    .................................aguggagagaguuuuaucucau......................................................   1       0    Fm3
    .................................aguggagagaguuuuaucucauc.....................................................   38      0    Fm3
    .................................aguggagagaguuuuaucucaucU....................................................   2       1    Fm3
    ................................................aucucaucgucugauauguuggguga....................................   5       0    Fm3
    .....................................................................Cugagauucaacuccuccaacug.................   2       1    Fm3
    .....................................................................gugagauucaacuccuccaacugc................   1       0    Fm3
    .....................................................................gugagauucaacucAuccaacugc................   1       1    Fm3
    ......................................................................ugagauucaacuccuccaa....................   18      0    Fm3
    ......................................................................ugagauucaacuccuccaU....................   1       1    Fm3
    ......................................................................ugagauucaacuccuccaaU...................   3       1    Fm3
    ......................................................................ugagauucaacuccuccaaA...................   2       1    Fm3
    ......................................................................ugagauucaacuccuccaac...................   45      0    Fm3
    ......................................................................ugagauucaaAuccuccaac...................   1       1    Fm3
    ......................................................................ugUgauucaacuccuccaacu..................   1       1    Fm3
    ......................................................................ugagauucaaUuccuccaacu..................   1       1    Fm3
    ......................................................................ugagauucaacuccuccGacu..................   5       1    Fm3
    ......................................................................ugagauucaacuccuccUacu..................   8       1    Fm3
    ......................................................................ugagauucaacuccuccCacu..................   7       1    Fm3
    ......................................................................ugagauucaacucGuccaacu..................   1       1    Fm3
    ......................................................................ugagauucGacuccuccaacu..................   1       1    Fm3
    ......................................................................ugagauucaaAuccuccaacu..................   2       1    Fm3
    ......................................................................ugagauucaacuccucUaacu..................   6       1    Fm3
    ......................................................................ugagauucaacucAuccaacu..................   3       1    Fm3
    ......................................................................ugagauucaUcuccuccaacu..................   1       1    Fm3
    ......................................................................ugagauucaacucUuccaacu..................   1       1    Fm3
    ......................................................................ugagauucaaGuccuccaacu..................   4       1    Fm3
    ......................................................................ugagCuucaacuccuccaacu..................   1       1    Fm3
    ......................................................................ugagauucaacuccuccaacA..................   8       1    Fm3
    ......................................................................ugagauucaacuccucGaacu..................   3       1    Fm3
    ......................................................................Ngagauucaacuccuccaacu..................   1       1    Fm3
    ......................................................................ugagauucaacuccuccaacu..................   1536    0    Fm3
    ......................................................................ugagauuUaacuccuccaacu..................   1       1    Fm3
    ......................................................................ugagauucaacuccucAaacu..................   10      1    Fm3
```

Star Mature

ggcuccuucccuucuuacugacacaucaagagaaguggagagaguuuuaucucaucgucugauauguuggugagauucaacuccuccaacugcucucggugugaaccau

....ugagauAcaacuccuccaacug.................	3	1	Fm3
....ugaAauucaacuccuccaacug.................	3	1	Fm3
....ugagauuAaacuccuccaacug.................	3	1	Fm3
....ugagauucaacuccuccaacug.................	19620	0	Fm3
....ugagauucaacAccuccaacug.................	3	1	Fm3
....uCagauucaacuccuccaacug.................	4	1	Fm3
....ugagCuucaacuccuccaacug.................	26	1	Fm3
....ugagauucCacuccuccaacug.................	1	1	Fm3
....ugagauucaacuUcuccaacug.................	1	1	Fm3
....ugagauucaacuccuccUacug.................	58	1	Fm3
....ugGgauucaacuccuccaacug.................	7	1	Fm3
....ugagaCucaacuccuccaacug.................	2	1	Fm3
....ugagauucaacuccuGcaacug.................	1	1	Fm3
....ugCgauucaacuccuccaacug.................	2	1	Fm3
....ugagauuUaacuccuccaacug.................	7	1	Fm3
....ugagauucaacuccuccaacuA.................	8	1	Fm3
....ugUgauucaacuccuccaacug.................	6	1	Fm3
....ugagauucaacuAcuccaacug.................	4	1	Fm3
....ugagauucaacuccuccaacuC.................	1	1	Fm3
....ugagauucaacuccCccaacug.................	2	1	Fm3
....ugagauCcaacuccuccaacug.................	2	1	Fm3
....Ngagauucaacuccuccaacug.................	8	1	Fm3
....ugagauucaacucAuccaacug.................	7	1	Fm3
....ugagauucaacuccucUaacug.................	52	1	Fm3
....ugagUuucaacuccuccaacug.................	5	1	Fm3
....ugagauucaacuccucAaacug.................	86	1	Fm3
....ugagauucaacGccuccaacug.................	2	1	Fm3
....ugagauucaacuccuccaUcug.................	2	1	Fm3
....ugagauucaacuccuccCacug.................	60	1	Fm3
....ugagauucaaAuccuccaacug.................	31	1	Fm3
....ugagauucaacuGcuccaacug.................	2	1	Fm3
....ugagauucUacuccuccaacug.................	10	1	Fm3
....ugagaAucaacuccuccaacug.................	2	1	Fm3
....Cgagauucaacuccuccaacug.................	4	1	Fm3
....ugagauucaacuccucGaacug.................	30	1	Fm3
....uUagauucaacuccuccaacug.................	7	1	Fm3
....ugagauucaacuccuccaacuU.................	7	1	Fm3
....ugagauucaacucUuccaacug.................	1	1	Fm3
....ugagauucaacuccuccaaAug.................	1	1	Fm3
....ugagGuucaacuccuccaacug.................	9	1	Fm3
....ugagauuGaacuccuccaacug.................	1	1	Fm3
....ugagauucaaGuccuccaacug.................	33	1	Fm3
....ugagauucGacuccuccaacug.................	3	1	Fm3
....ugagauucaacuccuccaacCg.................	2	1	Fm3
....ugagauucaacCccuccaacug.................	4	1	Fm3
....ugagauucaacuccuUcaacug.................	14	1	Fm3
....ugagauucaacuccuccaGcug.................	2	1	Fm3
....ugagauGcaacuccuccaacug.................	2	1	Fm3
....ugagauucaacuccuccaCcug.................	2	1	Fm3
....ugagauucaacuccuAcaacug.................	9	1	Fm3
....ugagauucaacuccuccGacug.................	85	1	Fm3
....ugaUauucaacuccuccaacug.................	1	1	Fm3
....uAagauucaacuccuccaacug.................	8	1	Fm3
....ugagauucaaUuccuccaacug.................	17	1	Fm3
....ugagauucaUcuccuccaacug.................	3	1	Fm3
....ugagauucaacuccAccaacug.................	1	1	Fm3
....ugagauuAaacuccuccaacugc................	1	1	Fm3
....ugagauucaacuccucGaacugc................	19	1	Fm3
....ugagaNucaacuccuccaacugc................	4	1	Fm3
....ugUgauucaacuccuccaacugc................	2	1	Fm3
....ugagauucaacuccuccaacugc................	10335	0	Fm3
....ugagauCcaacuccuccaacugc................	1	1	Fm3
....ugagauucaacAccuccaacugc................	1	1	Fm3
....ugagauucaacuAcuccaacugc................	3	1	Fm3
....ugagauucaacuccuccaacugU................	4628	1	Fm3
....ugagauAcaacuccuccaacugc................	3	1	Fm3
....ugagauucaacuccAccaacugc................	1	1	Fm3
....ugagauucGacuccuccaacugc................	2	1	Fm3
....ugagauucaaGuccuccaacugc................	12	1	Fm3
....ugagauucaacucAuccaacugc................	5	1	Fm3

Star Mature

```
ggcuccuucccuucuuacugacacaucaagagaaguggagagaguuuuaucucaucgucugauauguuggugagauucaacuccuccaacugcucucggugugaaccau
......................................................................uAagauucaacuccuccaacugc................ 5 1 Fm3
......................................................................ugagauucaacuccuUcaacugc................ 1 1 Fm3
......................................................................ugagauucaacuccuccUacugc................ 38 1 Fm3
......................................................................uUagauucaacuccuccaacugc................ 4 1 Fm3
......................................................................ugagauucaacuccuccaUcugc................ 1 1 Fm3
......................................................................Ggagauucaacuccuccaacugc................ 1 1 Fm3
......................................................................Ngagauucaacuccuccaacugc................ 4 1 Fm3
......................................................................ugagauucaacuccuAcaacugc................ 6 1 Fm3
......................................................................ugGgauucaacuccuccaacugc................ 3 1 Fm3
......................................................................Agagauucaacuccuccaacugc................ 1 1 Fm3
......................................................................ugagauucaGcuccuccaacugc................ 2 1 Fm3
......................................................................ugagauucaacuccuccCacugc................ 26 1 Fm3
......................................................................ugagauucaacuUcuccaacugc................ 3 1 Fm3
......................................................................ugagauucaacuccuccaacugG................ 5 1 Fm3
......................................................................ugagauucaacuccuccaacuAc................ 3 1 Fm3
......................................................................ugagauucaacuccCccaacugc................ 1 1 Fm3
......................................................................ugagCuucaacuccuccaacugc................ 10 1 Fm3
......................................................................Cgagauucaacuccuccaacugc................ 1 1 Fm3
......................................................................ugagauucaaUuccuccaacugc................ 5 1 Fm3
......................................................................ugagauucaacuccuccaGcugc................ 2 1 Fm3
......................................................................ugagGuucaacuccuccaacugc................ 1 1 Fm3
......................................................................ugagauucaacuccucUaacugc................ 23 1 Fm3
......................................................................ugagaAucaacuccuccaacugc................ 4 1 Fm3
......................................................................ugagauucaacuccuccaacugA................ 75 1 Fm3
......................................................................uNagauucaacuccuccaacugc................ 1 1 Fm3
......................................................................ugagauucaaAuccuccaacugc................ 17 1 Fm3
......................................................................ugagauucUacuccuccaacugc................ 8 1 Fm3
......................................................................ugagauucaacuGcuccaacugc................ 2 1 Fm3
......................................................................ugagaCucaacuccuccaacugc................ 2 1 Fm3
......................................................................ugagauuUaacuccuccaacugc................ 2 1 Fm3
......................................................................ugagauucaacCccuccaacugc................ 4 1 Fm3
......................................................................ugagauucaacuccucAaacugc................ 46 1 Fm3
......................................................................ugagauucaacuccuGcaacugc................ 1 1 Fm3
......................................................................ugagauucaUcuccuccaacugc................ 1 1 Fm3
......................................................................ugagauucaacuccuccGacugc................ 38 1 Fm3
......................................................................ugagauucCacuccuccaacugc................ 3 1 Fm3
......................................................................ugagauucaacuccuccaacugcC............... 4 1 Fm3
......................................................................ugagauucaacuccucGaacugcu............... 2 1 Fm3
......................................................................Cgagauucaacuccuccaacugcu............... 1 1 Fm3
......................................................................ugagauucaacuccuccUacugcu............... 1 1 Fm3
......................................................................ugagauucaacuccuccGacugcu............... 2 1 Fm3
......................................................................ugagauNcaacuccuccaacugcu............... 1 1 Fm3
......................................................................ugagauucaacuccuccaacugcu............... 432 0 Fm3
......................................................................ugagauucaacuccuccaacugcA............... 196 1 Fm3
......................................................................ugagauucaacuccuccaacugcG............... 2 1 Fm3
......................................................................ugagauucaacuccuccCacugcu............... 2 1 Fm3
......................................................................ugagauucaacuccuccaacugUu............... 90 1 Fm3
......................................................................ugagauucaacuccucUaacugcu............... 2 1 Fm3
......................................................................ugagauucaacuccucAaacugcu............... 1 1 Fm3
.......................................................................gagauucaacuccuccaacu.................. 3 0 Fm3
.......................................................................gagauuAaacuccuccaacug................. 1 1 Fm3
.......................................................................gagauucaacuccuccaacug................. 20 0 Fm3
.......................................................................gagauucaacAccuccaacug................. 1 1 Fm3
.......................................................................gagauucaacuccuccaacuA................. 1 1 Fm3
.......................................................................gagauucaacuccuAcaacug................. 1 1 Fm3
.......................................................................gagauucaacuccuccaacugU................ 5 1 Fm3
.......................................................................gagauucaacuccuccaacugc................ 5 0 Fm3
........................................................................agauucaacuccuccaacug................. 3 0 Fm3
........................................................................agauucaacuccuccaacugA................ 4 1 Fm3
........................................................................agauucaacuccuccaacugU................ 3 1 Fm3
........................................................................agauucaacuccuccaUcugc................ 1 1 Fm3
........................................................................agauucaacuccuccaacugc................ 71 0 Fm3
........................................................................agauucaacuccuccaacugcu............... 1 0 Fm3
........................................................................agauucaacuccuccaacugcA............... 1 1 Fm3
.........................................................................gauucaacuccuccaacug................. 17 0 Fm3
.........................................................................gauucaacuccuccaacugU................ 11 1 Fm3
.........................................................................gauucaacuccuccaacugc................ 1 0 Fm3
.........................................................................gauucaacuccuccaacugcu............... 2 0 Fm3
..........................................................................auucaacuccuccaacug................. 1 0 Fm3
..........................................................................auucaacuccuccaacugc................ 3 0 Fm3
```

```
                                 Star                                  Mature
ggcuccuucccuucuuacugacacaucaagagaaguggagagaguuuuaucucaucgucugauauguuggugagauucaacucccuccaacugcucucggugugaaccau
..........................................................................auucaacucccuccaacugU................ 1     1   Fm3
...........................................................................uucaacucccuccaacugc................ 5     0   Fm3
...........................................................................uucaacucccuccaacugU................ 7     1   Fm3
............................................................................ucaacucccuccaacugcC............... 1     1   Fm3
............................................................................ucaacucccuccaacugcu............... 1     0   Fm3

.................................aguggagagaguuuuaucu.......................................................... 1     0   Mm3
.................................aguggagagaguuuuaucucau....................................................... 3     0   Mm3
.................................aguggagagaguuuuaucucUuc...................................................... 1     1   Mm3
.................................aguggagagaguuuuaucucauU...................................................... 2     1   Mm3
.................................aguggagagaguuuuaucucauc...................................................... 23    0   Mm3
..................................guggagagaguuuuaucucauc...................................................... 1     0   Mm3
..............................................................uauguuggugagauucaacucccuU....................... 1     1   Mm3
.....................................................................Cugagauucaacucccuccaacu.................. 1     1   Mm3
.....................................................................Cugagauucaacucccuccaacug................. 17    1   Mm3
.....................................................................Cugagauucaacucccuccaacugc................ 8     1   Mm3
.....................................................................Cugagauucaacucccuccaacugcu............... 2     1   Mm3
......................................................................ugagauucaacucccucca..................... 3     0   Mm3
......................................................................ugagauucaacucccuccaa.................... 57    0   Mm3
......................................................................ugagUuucaacucccuccaa.................... 1     1   Mm3
......................................................................ugagauucaacucccuccaU.................... 15    1   Mm3
......................................................................Cgagauucaacucccuccaac................... 1     1   Mm3
......................................................................ugagauucGacucccuccaac................... 1     1   Mm3
......................................................................ugagauucaacucccuccaac................... 187   0   Mm3
......................................................................ugUgauucaacucccuccaac................... 1     1   Mm3
......................................................................ugagauucaacucccuccaaU................... 4     1   Mm3
......................................................................ugagauucaacucccucAaac................... 2     1   Mm3
......................................................................ugagauucaacucccuccaacC.................. 5     1   Mm3
......................................................................ugagauucaacucccuccaaAu.................. 1     1   Mm3
......................................................................ugagauucaacucccuccaaUu.................. 3     1   Mm3
......................................................................ugUgauucaacucccuccaacu.................. 1     1   Mm3
......................................................................ugagauucaaUucccuccaacu.................. 9     1   Mm3
......................................................................ugagauucaacuccCccaacu.................. 1     1   Mm3
......................................................................ugagauucaacucccuccaacG.................. 87    1   Mm3
......................................................................ugagauucaacucccuCcacu.................. 13    1   Mm3
......................................................................ugagauuAaacucccuccaacu.................. 1     1   Mm3
......................................................................ugagauucUacucccuccaacu.................. 1     1   Mm3
......................................................................ugagauucaacucccucAaacu.................. 37    1   Mm3
......................................................................ugagCuucaacucccuccaacu.................. 4     1   Mm3
......................................................................ugaAauucaacucccuccaacu.................. 1     1   Mm3
......................................................................ugagauucaacucccuccaacA.................. 30    1   Mm3
......................................................................ugagauucaaAucccuccaacu.................. 8     1   Mm3
......................................................................Agagauucaacucccuccaacu.................. 1     1   Mm3
......................................................................ugagauucaacuGccuccaacu.................. 2     1   Mm3
......................................................................ugagauucaacucGuccaacu.................. 1     1   Mm3
......................................................................ugagauucaacCccuccaacu.................. 1     1   Mm3
......................................................................ugagauucaacucccuccGacu.................. 28    1   Mm3
......................................................................ugagauucGacucccuccaacu.................. 8     1   Mm3
......................................................................ugagauucaacucccucUaacu.................. 19    1   Mm3
......................................................................ugGgauucaacucccuccaacu.................. 3     1   Mm3
......................................................................ugagauucaacucccuccUacu.................. 25    1   Mm3
......................................................................ugagauucaaGucccuccaacu.................. 6     1   Mm3
......................................................................ugagaNucaacucccuccaacu.................. 2     1   Mm3
......................................................................ugagauucaacucccuccaacu.................. 6262  0   Mm3
......................................................................uNagauucaacucccuccaacu.................. 1     1   Mm3
......................................................................ugagauucaacGccuccaacu.................. 1     1   Mm3
......................................................................ugagauucaacucccuccaGcu.................. 1     1   Mm3
......................................................................ugaCauucaacucccuccaacu.................. 1     1   Mm3
......................................................................uAagauucaacucccuccaacu.................. 1     1   Mm3
......................................................................ugagauucaGcucccuccaacu.................. 2     1   Mm3
......................................................................ugagauucCacucccuccaacu.................. 1     1   Mm3
......................................................................ugCgauucaacucccuccaacu.................. 1     1   Mm3
......................................................................ugagUuucaacucccuccaacu.................. 2     1   Mm3
......................................................................ugagauucaacuccuUcaacu.................. 2     1   Mm3
......................................................................ugagauucaacucccucGaacu.................. 9     1   Mm3
......................................................................ugagauucaacucUuccaacu.................. 6     1   Mm3
......................................................................ugagauuUaacucccuccaacu.................. 4     1   Mm3
......................................................................ugagauucaacuccuAcaacu.................. 1     1   Mm3
......................................................................ugagCuucaacucccuccaacug................. 9     1   Mm3
......................................................................ugagauucaacucccuccaCcug................. 1     1   Mm3
```

```
                                 Star                                  Mature
ggcuccuucccuucuuacugacacaucaagagaaguggagagaguuuuaucucaucgucugauauguuggugagauucaacuccuccaacugcucucggugugaaccau
......................................................................ugNgauucaacuccuccaacug.................    1      1   Mm3
......................................................................uAagauucaacuccuccaacug.................    7      1   Mm3
......................................................................ugagauucaacuccuAcaacug.................    2      1   Mm3
......................................................................Cgagauucaacuccuccaacug.................    5      1   Mm3
......................................................................ugagauucaacuccucNaacug.................    1      1   Mm3
......................................................................ugagauucaacuccuGcaacug.................    2      1   Mm3
......................................................................ugagauucaacuccuccaacuU.................    37     1   Mm3
......................................................................ugagUuucaacuccuccaacug.................    2      1   Mm3
......................................................................ugagauucaacuccuccaacAg.................    2      1   Mm3
......................................................................ugagauucaUcuccuccaacug.................    2      1   Mm3
......................................................................ugagauucaacuccCccaacug.................    2      1   Mm3
......................................................................uUagauucaacuccuccaacug.................    9      1   Mm3
......................................................................ugagauucaacuccucAaacug.................    120    1   Mm3
......................................................................ugagauuGaacuccuccaacug.................    1      1   Mm3
......................................................................ugagauucaacuccuUcaacug.................    19     1   Mm3
......................................................................ugagaGucaacuccuccaacug.................    1      1   Mm3
......................................................................ugagauGcaacuccuccaacug.................    5      1   Mm3
......................................................................ugagauucaacucUuccaacug.................    11     1   Mm3
......................................................................Ggagauucaacuccuccaacug.................    1      1   Mm3
......................................................................ugagauucaacuccuccaacug.................    28334  0   Mm3
......................................................................ugagauCcaacuccuccaacug.................    2      1   Mm3
......................................................................Ngagauucaacuccuccaacug.................    9      1   Mm3
......................................................................ugagauucaacuUcuccaacug.................    6      1   Mm3
......................................................................ugagaAucaacuccuccaacug.................    1      1   Mm3
......................................................................ugagauucaacuccuccaacuA.................    60     1   Mm3
......................................................................ugagauucaCcuccuccaacug.................    2      1   Mm3
......................................................................ugagauucaacCccuccaacug.................    5      1   Mm3
......................................................................ugUgauucaacuccuccaacug.................    5      1   Mm3
......................................................................Agagauucaacuccuccaacug.................    4      1   Mm3
......................................................................ugagauucaacuccuccaGcug.................    5      1   Mm3
......................................................................ugaAauucaacuccuccaacug.................    3      1   Mm3
......................................................................ugagauucaacuccuccaacCg.................    10     1   Mm3
......................................................................ugagauucaaUuccuccaacug.................    27     1   Mm3
......................................................................ugagauAcaacuccuccaacug.................    2      1   Mm3
......................................................................ugagauucaacuccAccaacug.................    1      1   Mm3
......................................................................ugagauucaacuccuccUacug.................    77     1   Mm3
......................................................................ugagauucaaGuccuccaacug.................    42     1   Mm3
......................................................................ugagauucaacucAuccaacug.................    5      1   Mm3
......................................................................ugagauucaacuccuccaacGg.................    178    1   Mm3
......................................................................ugagauucaacuccuccGacug.................    114    1   Mm3
......................................................................uCagauucaacuccuccaacug.................    4      1   Mm3
......................................................................ugagauucaacuccucGaacug.................    43     1   Mm3
......................................................................ugagauucaacAccuccaacug.................    2      1   Mm3
......................................................................ugCgauucaacuccuccaacug.................    1      1   Mm3
......................................................................ugagauNcaacuccuccaacug.................    1      1   Mm3
......................................................................ugagauucaacuccuccaaUug.................    2      1   Mm3
......................................................................ugagauucaGcuccuccaacug.................    3      1   Mm3
......................................................................ugagauuUaacuccuccaacug.................    7      1   Mm3
......................................................................ugagauuAaacuccuccaacug.................    1      1   Mm3
......................................................................ugagGuucaacuccuccaacug.................    4      1   Mm3
......................................................................ugGgauucaacuccuccaacug.................    10     1   Mm3
......................................................................ugagauucaacuccuccaUcug.................    3      1   Mm3
......................................................................ugagaCucaacuccuccaacug.................    3      1   Mm3
......................................................................ugagauucGacuccuccaacug.................    7      1   Mm3
......................................................................ugagaNucaacuccuccaacug.................    5      1   Mm3
......................................................................ugagauucaacuccucUaacug.................    80     1   Mm3
......................................................................ugagauucUacuccuccaacug.................    9      1   Mm3
......................................................................ugagauucaacuccuccaaAug.................    1      1   Mm3
......................................................................ugagauucaacuccuccCacug.................    85     1   Mm3
......................................................................ugagauucaaAuccuccaacug.................    39     1   Mm3
......................................................................ugagaAucaacuccuccaacugc................    5      1   Mm3
......................................................................ugagauuAaacuccuccaacugc................    4      1   Mm3
......................................................................ugagauucCacuccuccaacugc................    14     1   Mm3
......................................................................Agagauucaacuccuccaacugc................    7      1   Mm3
......................................................................ugagauuUaacuccuccaacugc................    21     1   Mm3
......................................................................Cgagauucaacuccuccaacugc................    10     1   Mm3
......................................................................ugagauucaacuccucAaacugc................    287    1   Mm3
......................................................................ugagaGucaacuccuccaacugc................    1      1   Mm3
......................................................................ugagauucaacuccuccaacuAc................    14     1   Mm3
......................................................................ugagauucaacuccuccaacugU................    17627  1   Mm3
```

Star **Mature**

ggcuccuucccuucuuacugacacaucaagagaaguggagagaguuuuaucucaucgucugauauguuggugagauucaacuccuccaacugcucucggugugaaccau			
....ugagaCucaacuccuccaacugc................	9	1	Mm3
....ugagCuucaacuccuccaacugc................	73	1	Mm3
....ugagauucaacuccuccaacuCc................	2	1	Mm3
....ugNgauucaacuccuccaacugc................	2	1	Mm3
....ugagauucaacuccuccaaUugc................	10	1	Mm3
....ugagauucaacuccuccaacGgc................	3	1	Mm3
....ugagauucaacuccuccaacugG................	184	1	Mm3
....ugagauucaacuccucGaacugc................	97	1	Mm3
....ugagauucaacucUuccaacugc................	17	1	Mm3
....ugagauucaacuccuUcaacugc................	10	1	Mm3
....ugagauucaCcuccuccaacugc................	3	1	Mm3
....Ggagauucaacuccuccaacugc................	1	1	Mm3
....ugagauucaacuGcuccaacugc................	2	1	Mm3
....ugaCauucaacuccuccaacugc................	2	1	Mm3
....ugagauucaacuccuAcaacugc................	1	1	Mm3
....uAagauucaacuccuccaacugc................	37	1	Mm3
....ugagauucaGcuccuccaacugc................	10	1	Mm3
....ugagauucaacGccuccaacugc................	1	1	Mm3
....ugaAauucaacuccuccaacugc................	5	1	Mm3
....ugagauucaacuccuccCacugc................	216	1	Mm3
....uCagauucaacuccuccaacugc................	5	1	Mm3
....ugagauucaacCccuccaacugc................	5	1	Mm3
....uUagauucaacuccuccaacugc................	13	1	Mm3
....ugagauucaacucGuccaacugc................	3	1	Mm3
....ugagauucaacuccuccaUcugc................	8	1	Mm3
....ugagauucaacuccAccaacugc................	4	1	Mm3
....ugagauNcaacuccuccaacugc................	7	1	Mm3
....ugagauucaaUuccuccaacugc................	81	1	Mm3
....ugagauucaacuccucUaacugc................	216	1	Mm3
....ugagUuucaacuccuccaacugc................	17	1	Mm3
....ugagauucaacuccuccGacugc................	281	1	Mm3
....ugagauucaacuccuccaacugc................	72360	0	Mm3
....ugaUauucaacuccuccaacugc................	5	1	Mm3
....ugagauAcaacuccuccaacugc................	2	1	Mm3
....ugagauucaaGuccuccaacugc................	87	1	Mm3
....ugagGuucaacuccuccaacugc................	29	1	Mm3
....ugagauucUacuccuccaacugc................	64	1	Mm3
....ugagauucaacuccuccaacCgc................	7	1	Mm3
....ugagauucaaAuccuccaacugc................	104	1	Mm3
....ugagauucaacAccuccaacugc................	6	1	Mm3
....ugagauucaacuUcuccaacugc................	14	1	Mm3
....ugagauucaUcuccuccaacugc................	6	1	Mm3
....ugagauucaacuccuccaCcugc................	5	1	Mm3
....ugagauucaacuccuccaGcugc................	8	1	Mm3
....ugCgauucaacuccuccaacugc................	7	1	Mm3
....ugagaNucaacuccuccaacugc................	12	1	Mm3
....ugagauuGaacuccuccaacugc................	16	1	Mm3
....ugagauucaacuccuccaacugA................	231	1	Mm3
....ugagauGcaacuccuccaacugc................	2	1	Mm3
....ugagauucaacuAcuccaacugc................	6	1	Mm3
....Ngagauucaacuccuccaacugc................	34	1	Mm3
....ugagauCcaacuccuccaacugc................	9	1	Mm3
....ugagauucaacuccuccUacugc................	238	1	Mm3
....ugagauucaacuccuGcaacugc................	1	1	Mm3
....ugagauucaacuccGccaacugc................	1	1	Mm3
....ugagauuGaacuccuccaacugc................	4	1	Mm3
....ugagauucaacuccuccaaAugc................	2	1	Mm3
....ugagauucaacucAuccaacugc................	10	1	Mm3
....uNagauucaacuccuccaacugc................	4	1	Mm3
....ugUgauucaacuccuccaacugc................	10	1	Mm3
....ugagauucaacuccCccaacugc................	9	1	Mm3
....ugagauucaacuccuccaacuUc................	12	1	Mm3
....ugagauucaacuccuccaacAgc................	2	1	Mm3
....ugGgauucaacuccuccaacugc................	19	1	Mm3
....ugagauucaacuccucGaacugcu...............	4	1	Mm3
....ugagauucaaAuccuccaacugcu...............	9	1	Mm3
....ugagauucaGcuccuccaacugcu...............	1	1	Mm3
....ugagauucaacuccuAcaacugcu...............	1	1	Mm3
....ugGgauucaacuccuccaacugcu...............	2	1	Mm3
....ugagauucaacuccCccaacugcu...............	4	1	Mm3

```
                                Star                              Mature
ggcuccuucccuucuuacugacacaucaagagaaguggagagaguuuuaucucaucgucugauauguuggugagauucaacuccuccaacugcucucggugugaaccau
......................................................................ugagauucaacuccAccaacugcu...............    1     1     Mm3
......................................................................ugagauucaacuccuccaacuUcu...............    1     1     Mm3
......................................................................ugagauuUaacuccuccaacugcu...............    1     1     Mm3
......................................................................ugagauucaaUuccuccaacugcu...............    9     1     Mm3
......................................................................ugagauucaacuUcuccaacugcu...............    1     1     Mm3
......................................................................ugagauucaacuccuccCacugcu...............    15    1     Mm3
......................................................................ugagauucaacucUuccaacugcu...............    1     1     Mm3
......................................................................ugCgauucaacuccuccaacugcu...............    1     1     Mm3
......................................................................ugagauucaacuccuccUacugcu...............    21    1     Mm3
......................................................................ugagauucaacuccuccaaUugcu...............    1     1     Mm3
......................................................................ugagauucaacuccucUaacugcu...............    10    1     Mm3
......................................................................ugagaCucaacuccuccaacugcu...............    1     1     Mm3
......................................................................ugagauucaacuccuccaacugcG...............    57    1     Mm3
......................................................................ugagaNucaacuccuccaacugcu...............    1     1     Mm3
......................................................................ugagauucaacuccuccaacugcu...............    5015  0     Mm3
......................................................................ugaAauucaacuccuccaacugcu...............    1     1     Mm3
......................................................................ugagauucGacuccuccaacugcu...............    1     1     Mm3
......................................................................ugagauucaacCccuccaacugcu...............    3     1     Mm3
......................................................................uAagauucaacuccuccaacugcu...............    1     1     Mm3
......................................................................ugagauucaacuccuccaacugAu...............    5     1     Mm3
......................................................................ugagauucaacuccuccaacugcC...............    12    1     Mm3
......................................................................ugagauucaacuccuccaacugcA...............    861   1     Mm3
......................................................................ugagauucCacuccuccaacugcu...............    1     1     Mm3
......................................................................ugagauucaacuccuccGacugcu...............    19    1     Mm3
......................................................................uUagauucaacuccuccaacugcu...............    2     1     Mm3
......................................................................ugagaAucaacuccuccaacugcu...............    1     1     Mm3
......................................................................ugagauucUacuccuccaacugcu...............    2     1     Mm3
......................................................................ugagUuucaacuccuccaacugcu...............    2     1     Mm3
......................................................................ugagauucaacuccuccaacugUu...............    619   1     Mm3
......................................................................ugagGuucaacuccuccaacugcu...............    1     1     Mm3
......................................................................ugagauucaacucAuccaacugcu...............    2     1     Mm3
......................................................................ugagCuucaacuccuccaacugcu...............    1     1     Mm3
......................................................................ugagauucaaGuccuccaacugcu...............    4     1     Mm3
......................................................................ugagauAcaacuccuccaacugcu...............    1     1     Mm3
......................................................................uCagauucaacuccuccaacugcu...............    1     1     Mm3
......................................................................ugagauucaacuccuccaacCgcu...............    2     1     Mm3
......................................................................ugagauucaacuccucAaacugcu...............    19    1     Mm3
......................................................................Ngagauucaacuccuccaacugcu...............    5     1     Mm3
......................................................................ugagauucaacuccuUcaacugcu...............    1     1     Mm3
......................................................................Agagauucaacuccuccaacugcu...............    1     1     Mm3
......................................................................ugagauucaacuccuccaUcugcu...............    1     1     Mm3
......................................................................ugagauucaacuccuccaacugcuc..............    1     0     Mm3
......................................................................ugagauucaacuccuccaacugcuA..............    3     1     Mm3
......................................................................ugagauucaacuccuccaacugUuc..............    1     1     Mm3
......................................................................ugagauucaacuccuccaacugcuGu.............    1     1     Mm3
......................................................................ugagauucaacuccuccaacugcuAu.............    2     1     Mm3
.......................................................................gagauucaacAccuccaacu..................    1     1     Mm3
.......................................................................gagauucaacuccuccaacu..................    7     0     Mm3
.......................................................................gagauucaacuccuccaacug.................    40    0     Mm3
.......................................................................gagauucaacuccuAcaacug.................    1     1     Mm3
.......................................................................gagauucaacuccuccaacugU................    26    1     Mm3
.......................................................................gagauucaacuccuccaacugG................    1     1     Mm3
.......................................................................Uagauucaacuccuccaacugc................    3     1     Mm3
.......................................................................gagauucaacuccuccaacugc................    20    0     Mm3
.......................................................................gagauucaacuccuccaacCgc................    1     1     Mm3
.......................................................................gagauucaacuccuccaacugcu...............    3     0     Mm3
.......................................................................gagauucaacuccuccaacugUu...............    1     1     Mm3
........................................................................agauucaacuccuccaacu..................    6     0     Mm3
........................................................................agauucaacuccuccaacug.................    6     0     Mm3
........................................................................agauucaacuccuccaacugc................    159   0     Mm3
........................................................................agauucaacuccuccaacugU................    18    1     Mm3
........................................................................Ugauucaacuccuccaacugc................    4     1     Mm3
........................................................................agauucaacuGcuccaacugc................    1     1     Mm3
........................................................................agauucaacuccuccaaAugc................    2     1     Mm3
........................................................................agauucaacuccuccaaUugc................    1     1     Mm3
........................................................................agauucaacuccuccaacugcA...............    6     1     Mm3
........................................................................agauucaacuccuccaacugcu...............    20    0     Mm3
.........................................................................gauucaacuccuccaacug.................    6     0     Mm3
.........................................................................gauucaacuccuccaacugG................    1     1     Mm3
.........................................................................gauucaacuccuccaacugU................    12    1     Mm3
```

Star Mature

```
ggcuccuucccuucuuacugacacaucaagagaaguggagagaguuuuaucucaucgucugauauguuggugagauucaacuccuccaacugcucucggugugaaccau
.........................................................................gauucaacuccuccaacugUu...............  1    1  Mm3
..........................................................................auucaacuccuccaacug.................  4    0  Mm3
..........................................................................auucaacuccuccaacugc................  1    0  Mm3
..........................................................................auucaacuccuccaacugU................  3    1  Mm3
..........................................................................auucaacuccuccaacugG................  1    1  Mm3
..........................................................................auucaacuccuccaacugcG...............  1    1  Mm3
...........................................................................uucaacuccuccaacugU................  6    1  Mm3
...........................................................................uucaacuccuccaacugc................  5    0  Mm3

.....cuucccuucuuacugacacaucaagaga............................................................................  3    0  Fm1
.............cuuacugacacauUaagaga............................................................................  1    1  Fm1
.............cuuacugacacaucaagaga............................................................................  3    0  Fm1
.................................aguggagagaguuuuauc..........................................................  6    0  Fm1
.................................aguggagagaguuuuaucu.........................................................  1    0  Fm1
.................................aguggagagaguuuuaucuc........................................................  1    0  Fm1
.................................aguggagagaguuuuaucuca.......................................................  7    0  Fm1
.................................aguggagagaguuuuaucucau......................................................  7    0  Fm1
.................................aguggagagaguuuuaucucauU.....................................................  19   1  Fm1
.................................aguggagagagGuuuaucucauc.....................................................  1    1  Fm1
.................................aguggagagaguuuuaAcucauc.....................................................  1    1  Fm1
.................................aguggagagaguuuuauAucauc.....................................................  7    1  Fm1
.................................aguggagagaguuuuaGcucauc.....................................................  1    1  Fm1
.................................aguggagUgaguuuuaucucauc.....................................................  1    1  Fm1
.................................aguggagagaguuuuaucucauc.....................................................  465  0  Fm1
.................................aguggagagaguuuuaucucauG.....................................................  3    1  Fm1
.................................aguggagagGguuuuaucucauc.....................................................  1    1  Fm1
.................................aguggagagUguuuuaucucauc.....................................................  2    1  Fm1
.................................aguggagagaguuuuaCcucauc.....................................................  1    1  Fm1
.................................aguggagagaAuuuuaucucauc.....................................................  1    1  Fm1
.................................aguggagagaguuuuauGucauc.....................................................  2    1  Fm1
.................................aguggagagaguuuuauUucauc.....................................................  1    1  Fm1
.................................aguggagagCguuuuaucucauc.....................................................  1    1  Fm1
.................................aguggNgagaguuuuaucucauc.....................................................  1    1  Fm1
.................................aguggagagaguuuuaucucaucA....................................................  6    1  Fm1
.................................aguggagagaguuuuaucucaucU....................................................  3    1  Fm1
..................................guggagagaguuuuaucucauU.....................................................  1    1  Fm1
..................................guggagagaguuuuaucAcauc.....................................................  1    1  Fm1
..................................guggagagaguuuuaucucauc.....................................................  81   0  Fm1
..................................guggagagGuuuaucucauc.......................................................  1    1  Fm1
..................................gAggagagaguuuuaucucauc.....................................................  1    1  Fm1
..................................guggagagaguuuuaucucaucU....................................................  11   1  Fm1
...................................uggagagaguuuuaucucauc.....................................................  2    0  Fm1
...................................................................uggugagauucaacuccuccaU....................  3    1  Fm1
....................................................................ggugagauucaacuccuccaacugU................  5    1  Fm1
.....................................................................Cugagauucaacuccuccaacug.................  3    1  Fm1
.....................................................................Cugagauucaacuccuccaacugc................  16   1  Fm1
......................................................................ugagauucaacuccuccU.....................  16   1  Fm1
......................................................................ugagauucaacuccucca.....................  32   0  Fm1
......................................................................ugagauucaacuccucUa.....................  1    1  Fm1
......................................................................ugGgauucaacuccuccaa....................  1    1  Fm1
......................................................................ugagauucaacuccucUaa....................  1    1  Fm1
......................................................................ugagauucaacuccuccaU....................  40   1  Fm1
......................................................................ugagauucaaAuccuccaa....................  1    1  Fm1
......................................................................ugagauucaacuccuccCa....................  2    1  Fm1
......................................................................ugagauucaacuccuccaa....................  459  0  Fm1
......................................................................ugagauucaacuccuccaG....................  2    1  Fm1
......................................................................ugagauucaCcuccuccaa....................  1    1  Fm1
......................................................................ugagauucaacuccucAaa....................  1    1  Fm1
......................................................................ugagauucaacuccucAaac...................  2    1  Fm1
......................................................................ugagauucaacuccucUaac...................  3    1  Fm1
......................................................................ugagauucaacuccuccGac...................  1    1  Fm1
......................................................................ugagauucaacuccuccaac...................  462  0  Fm1
......................................................................ugagauucaacuccuccaaU...................  13   1  Fm1
......................................................................ugagCuucaacuccuccaac...................  1    1  Fm1
......................................................................ugagauucaaGuccuccaac...................  3    1  Fm1
......................................................................ugagauucaacuccuccaaA...................  12   1  Fm1
......................................................................ugagauucUacuccuccaac...................  1    1  Fm1
......................................................................ugagauucaacuccuccaacG..................  232  1  Fm1
......................................................................ugagauuAaacuccuccaacu..................  1    1  Fm1
......................................................................ugagauucaacuccucUaacu..................  36   1  Fm1
```

```
                                 Star                       Mature
ggcuccuucccuucuuacugacacaucaagagaaguggagagaguuuuaucucaucgucugauauguuggugagauucaacuccuccaacugcucucggugugaaccau
......................................................................ugagauucCacuccuccaacu..................  1     1    Fm1
......................................................................ugagauCcaacuccuccaacu..................  1     1    Fm1
......................................................................ugagauucaacuccuccaCcu..................  1     1    Fm1
......................................................................ugagauucaacuccucGaacu..................  8     1    Fm1
......................................................................Agagauucaacuccuccaacu..................  2     1    Fm1
......................................................................ugagauucaacGccuccaacu..................  1     1    Fm1
......................................................................ugagaNucaacuccuccaacu..................  2     1    Fm1
......................................................................ugagauucaacucAuccaacu..................  3     1    Fm1
......................................................................uAagauucaacuccuccaacu..................  4     1    Fm1
......................................................................ugagauucaaAuccuccaacu..................  10    1    Fm1
......................................................................ugagauucaaGuccuccaacu..................  14    1    Fm1
......................................................................ugGgauucaacuccuccaacu..................  4     1    Fm1
......................................................................ugagauucaacuccuccaaGu..................  1     1    Fm1
......................................................................ugagauuUaacuccuccaacu..................  5     1    Fm1
......................................................................ugagauucaacuccuccaUcu..................  1     1    Fm1
......................................................................ugagauucUacuccuccaacu..................  5     1    Fm1
......................................................................ugagauucaacuccuUcaacu..................  4     1    Fm1
......................................................................ugagCuucaacuccuccaacu..................  2     1    Fm1
......................................................................ugagauucaaUuccuccaacu..................  7     1    Fm1
......................................................................ugagauucaUcuccuccaacu..................  2     1    Fm1
......................................................................ugagauucaacuccucAaacu..................  42    1    Fm1
......................................................................ugagauucaacCccuccaacu..................  1     1    Fm1
......................................................................ugagauucaacuccuccaacA..................  19    1    Fm1
......................................................................uCagauucaacuccuccaacu..................  1     1    Fm1
......................................................................ugagauucaCcuccuccaacu..................  1     1    Fm1
......................................................................ugagauuGaacuccuccaacu..................  1     1    Fm1
......................................................................Ngagauucaacuccuccaacu..................  5     1    Fm1
......................................................................ugagauucaacucUuccaacu..................  2     1    Fm1
......................................................................ugagauucGacuccuccaacu..................  2     1    Fm1
......................................................................ugagauucaacuccuccUacu..................  29    1    Fm1
......................................................................ugagauucaacuccuccGacu..................  41    1    Fm1
......................................................................ugagGuucaacuccuccaacu..................  2     1    Fm1
......................................................................ugagauucaacuGcuccaacu..................  1     1    Fm1
......................................................................ugagauucaacuccuccaacu..................  8524  0    Fm1
......................................................................ugagauucaacuccuAcaacu..................  1     1    Fm1
......................................................................ugagauucaacuccuccCacu..................  21    1    Fm1
......................................................................Cgagauucaacuccuccaacu..................  5     1    Fm1
......................................................................ugagauucaacuccuccaaAu..................  7     1    Fm1
......................................................................uNagauucaacuccuccaacu..................  2     1    Fm1
......................................................................ugagauucaacuccuccaaUu..................  3     1    Fm1
......................................................................ugagauucaacuccuccaacC..................  1     1    Fm1
......................................................................ugagauucaacuccuccCacug.................  22    1    Fm1
......................................................................ugagauucaacuUcuccaacug.................  2     1    Fm1
......................................................................ugagauucaacucAuccaacug.................  4     1    Fm1
......................................................................ugagauucaaGuccuccaacug.................  15    1    Fm1
......................................................................ugagauucaacuccucUaacug.................  32    1    Fm1
......................................................................Ggagauucaacuccuccaacug.................  1     1    Fm1
......................................................................ugagauucaacuccuccGacug.................  33    1    Fm1
......................................................................uUagauucaacuccuccaacug.................  2     1    Fm1
......................................................................ugaCauucaacuccuccaacug.................  1     1    Fm1
......................................................................ugagauucaacuccGccaacug.................  1     1    Fm1
......................................................................ugagauuAaacuccuccaacug.................  1     1    Fm1
......................................................................ugagauucUacuccuccaacug.................  3     1    Fm1
......................................................................ugagauAcaacuccuccaacug.................  1     1    Fm1
......................................................................uCagauucaacuccuccaacug.................  1     1    Fm1
......................................................................ugagauucaacuccuccaGcug.................  1     1    Fm1
......................................................................ugagauucaacuccuUcaacug.................  3     1    Fm1
......................................................................ugagCuucaacuccuccaacug.................  6     1    Fm1
......................................................................ugagauucaacuccuccaUcug.................  1     1    Fm1
......................................................................ugagauucaacuccuccaacCg.................  2     1    Fm1
......................................................................Cgagauucaacuccuccaacug.................  3     1    Fm1
......................................................................ugagauNcaacuccuccaacug.................  2     1    Fm1
......................................................................ugagauucaacuccuccaacug.................  8544  0    Fm1
......................................................................uAagauucaacuccuccaacug.................  6     1    Fm1
......................................................................ugagauucaacuccuccaacuC.................  1     1    Fm1
......................................................................ugGgauucaacuccuccaacug.................  5     1    Fm1
......................................................................ugagauucaacGccuccaacug.................  1     1    Fm1
......................................................................ugCgauucaacuccuccaacug.................  2     1    Fm1
......................................................................Ngagauucaacuccuccaacug.................  4     1    Fm1
......................................................................ugagGuucaacuccuccaacug.................  2     1    Fm1
```

```
                                 Star                                     Mature
ggcuccuucccuucuuacugacacaucaagagaaguggagagaguuuuaucucaucgucugauauguuggugagauucaacuccuccaacugcucucggugugaaccau
......................................................................ugagauucCacuccuccaacug.................  1      1  Fm1
......................................................................ugagaNucaacuccuccaacug.................  2      1  Fm1
......................................................................ugagauucaacuccuccaacuA.................  23     1  Fm1
......................................................................ugagauucaacuccucAaacug.................  46     1  Fm1
......................................................................ugagauucaacuAcuccaacug.................  1      1  Fm1
......................................................................ugagauucaacucUuccaacug.................  3      1  Fm1
......................................................................ugagauucaacuccuccaacGg.................  151    1  Fm1
......................................................................ugagauCcaacuccuccaacug.................  1      1  Fm1
......................................................................ugagauucaaUuccuccaacug.................  3      1  Fm1
......................................................................ugagauucaacuccuccaacuU.................  36     1  Fm1
......................................................................ugagauuUaacuccuccaacug.................  10     1  Fm1
......................................................................ugagauucaUcuccuccaacug.................  2      1  Fm1
......................................................................ugagauucaacuccuccUacug.................  36     1  Fm1
......................................................................ugagauucaaAuccuccaacug.................  18     1  Fm1
......................................................................ugagauucaacuccCccaacug.................  3      1  Fm1
......................................................................ugagauucaacuccucGaacug.................  14     1  Fm1
......................................................................ugagauucaacuccuccaaUug.................  3      1  Fm1
......................................................................ugagauucaacuccuccaacAg.................  1      1  Fm1
......................................................................ugagauucaacuccuccaacugc................  49420  0  Fm1
......................................................................ugagauuUaacuccuccaacugc................  20     1  Fm1
......................................................................ugagauucaUcuccuccaacugc................  4      1  Fm1
......................................................................ugagauucaacuGcuccaacugc................  5      1  Fm1
......................................................................ugagauucaacuccuccaCcugc................  3      1  Fm1
......................................................................ugagauucaacuccucGaacugc................  70     1  Fm1
......................................................................ugagauucaacuccAccaacugc................  1      1  Fm1
......................................................................ugagauuAaacuccuccaacugc................  1      1  Fm1
......................................................................uUagauucaacuccuccaacugc................  6      1  Fm1
......................................................................ugagCuucaacuccuccaacugc................  21     1  Fm1
......................................................................ugagauucaacuccuUcaacugc................  26     1  Fm1
......................................................................ugagauucaacuccucAaacugc................  213    1  Fm1
......................................................................ugagauCcaacuccuccaacugc................  3      1  Fm1
......................................................................ugaAauucaacuccuccaacugc................  2      1  Fm1
......................................................................ugagauucaacAccuccaacugc................  3      1  Fm1
......................................................................ugagauucaacGccuccaacugc................  3      1  Fm1
......................................................................ugagaAucaacuccuccaacugc................  4      1  Fm1
......................................................................ugagauucaaGuccuccaacugc................  57     1  Fm1
......................................................................ugagauucaacucUuccaacugc................  11     1  Fm1
......................................................................ugagaCucaacuccuccaacugc................  4      1  Fm1
......................................................................ugagauucaacuccuccaaUugc................  14     1  Fm1
......................................................................ugNgauucaacuccuccaacugc................  1      1  Fm1
......................................................................uAagauucaacuccuccaacugc................  24     1  Fm1
......................................................................ugagauucaaAuccuccaacugc................  74     1  Fm1
......................................................................ugagauucaacuccuccaacugA................  79     1  Fm1
......................................................................Ngagauucaacuccuccaacugc................  17     1  Fm1
......................................................................ugagauAcaacuccuccaacugc................  5      1  Fm1
......................................................................ugaUauucaacuccuccaacugc................  2      1  Fm1
......................................................................ugagGuucaacuccuccaacugc................  6      1  Fm1
......................................................................ugCgauucaacuccuccaacugc................  3      1  Fm1
......................................................................ugUgauucaacuccuccaacugc................  10     1  Fm1
......................................................................ugagauucaacuccuccaacuAc................  7      1  Fm1
......................................................................ugagauucaacuccuAcaacugc................  4      1  Fm1
......................................................................ugagauucaacuccuccaUcugc................  6      1  Fm1
......................................................................ugagauucaacuccuccaacuUc................  7      1  Fm1
......................................................................ugagauGcaacuccuccaacugc................  2      1  Fm1
......................................................................ugagauuGaacuccuccaacugc................  3      1  Fm1
......................................................................ugagauucCacuccuccaacugc................  4      1  Fm1
......................................................................ugagauucaacCccuccaacugc................  10     1  Fm1
......................................................................ugagauucaacuccucUaacugc................  134    1  Fm1
......................................................................ugagauucaacuccuccaacugU................  6115   1  Fm1
......................................................................ugagauucaacuccCccaacugc................  7      1  Fm1
......................................................................ugagaGucaacuccuccaacugc................  1      1  Fm1
......................................................................ugagauucaacuccuccaacuCc................  1      1  Fm1
......................................................................Cgagauucaacuccuccaacugc................  15     1  Fm1
......................................................................ugagauucaacuUcuccaacugc................  18     1  Fm1
......................................................................uCagauucaacuccuccaacugc................  1      1  Fm1
......................................................................ugaCauucaacuccuccaacugc................  4      1  Fm1
......................................................................ugagauucaacuccuGcaacugc................  2      1  Fm1
......................................................................ugagUuucaacuccuccaacugc................  3      1  Fm1
......................................................................ugagauucaacuccuccaacugG................  156    1  Fm1
......................................................................ugagauucaacuccuccaacAgc................  2      1  Fm1
```

Star Mature

ggcuccuucccuucuuacugacacaucaagagaaguggagagaguuuuaucucaucgucugauauguuggugagauucaacuccuccaacugcucucggugugaaccau			
....ugagauucGacuccuccaacugc................	19	1	Fm1
....ugagauucaacuccuccCacugc................	140	1	Fm1
....ugagaNucaacuccuccaacugc................	8	1	Fm1
....ugagauucaGcuccuccaacugc................	2	1	Fm1
....ugagauucaacuAcuccaacugc................	4	1	Fm1
....ugagauucaCcuccuccaacugc................	2	1	Fm1
....ugGgauucaacuccuccaacugc................	10	1	Fm1
....ugagauucaacuccuccGacugc................	188	1	Fm1
....ugagauucaacuccuccaGcugc................	10	1	Fm1
....Agagauucaacuccuccaacugc................	5	1	Fm1
....ugagauucaacucAuccaacugc................	9	1	Fm1
....Ggagauucaacuccuccaacugc................	2	1	Fm1
....ugagauucaacuccuccUacugc................	153	1	Fm1
....ugagauucaaUuccuccaacugc................	41	1	Fm1
....ugagauucUacuccuccaacugc................	29	1	Fm1
....ugagauNcaacuccuccaacugc................	1	1	Fm1
....ugagauucaacuccuccaacCgc................	4	1	Fm1
....ugagauucaacuccuccaacugcA...............	455	1	Fm1
....ugGgauucaacuccuccaacugcu...............	3	1	Fm1
....ugagauucaacuccuccaacugUu...............	293	1	Fm1
....ugagauucaacuccuccaacugGu...............	3	1	Fm1
....ugagauucUacuccuccaacugcu...............	2	1	Fm1
....Cgagauucaacuccuccaacugcu...............	1	1	Fm1
....ugagUuucaacuccuccaacugcu...............	2	1	Fm1
....ugagauucaacuGcuccaacugcu...............	2	1	Fm1
....ugagauucaacuccuccaacCgcu...............	1	1	Fm1
....Ngagauucaacuccuccaacugcu...............	4	1	Fm1
....ugagauucaaUuccuccaacugcu...............	4	1	Fm1
....ugagauucaGcuccuccaacugcu...............	1	1	Fm1
....ugagauucaacCccuccaacugcu...............	4	1	Fm1
....ugagaGucaacuccuccaacugcu...............	1	1	Fm1
....ugagCuucaacuccuccaacugcu...............	3	1	Fm1
....uCagauucaacuccuccaacugcu...............	1	1	Fm1
....ugagauucaacuccuccaGcugcu...............	1	1	Fm1
....ugagauucaacuccuccaaUugcu...............	9	1	Fm1
....ugagauucaacuccuccCacugcu...............	12	1	Fm1
....ugagauucaaGuccuccaacugcu...............	5	1	Fm1
....ugagauucaacuccuccaacugcC...............	3	1	Fm1
....ugagauucaacuccucAaacugcu...............	19	1	Fm1
....ugagauucaacuccuccaacuCcu...............	1	1	Fm1
....ugagauucaacuccAccaacugcu...............	2	1	Fm1
....ugagauucaaAuccuccaacugcu...............	11	1	Fm1
....ugagauucGacuccuccaacugcu...............	1	1	Fm1
....ugagauucaacuccucUaacugcu...............	16	1	Fm1
....ugCgauucaacuccuccaacugcu...............	1	1	Fm1
....uUagauucaacuccuccaacugcu...............	3	1	Fm1
....ugagauucCacuccuccaacugcu...............	2	1	Fm1
....ugagauucaacuccuccaacugcG...............	81	1	Fm1
....ugagauucaCcuccuccaacugcu...............	1	1	Fm1
....ugagauucaacuccuccUacugcu...............	15	1	Fm1
....ugagauucaacucAuccaacugcu...............	2	1	Fm1
....ugagauuUaacuccuccaacugcu...............	3	1	Fm1
....ugagaNucaacuccuccaacugcu...............	2	1	Fm1
....Agagauucaacuccuccaacugcu...............	1	1	Fm1
....ugagauucaacuccuccaacGgcu...............	1	1	Fm1
....ugagauucaacuccCccaacugcu...............	1	1	Fm1
....ugagauucaacuccuccaacugcu...............	4695	0	Fm1
....ugagauucaacuccuccGacugcu...............	12	1	Fm1
....ugagauuAaacuccuccaacugcu...............	1	1	Fm1
....ugagauNcaacuccuccaacugcu...............	1	1	Fm1
....ugagauucaacucUuccaacugcu...............	1	1	Fm1
....ugagauucaacuccucGaacugcu...............	4	1	Fm1
....uAagauucaacuccuccaacugcu...............	3	1	Fm1
....ugagauucaacuccuccaacuUcu...............	1	1	Fm1
....ugaUauucaacuccuccaacugcu...............	1	1	Fm1
....gagauucaacuccuccaacug................	6	0	Fm1
....gagauucaacuccuccaacugc...............	16	0	Fm1
....gagauucaacucAuccaacugc...............	1	1	Fm1
....gagauucaacuccuccaacugU...............	3	1	Fm1
....agauucaacuccuccaac..................	4	0	Fm1

Star Mature

```
ggcuccuucccuucuuacugacacaucaagagaaguggagagaguuuuaucucaucgucugauauguuggugagauucaacuccuccaacugcucucggugugaaccau
........................................................................agauucaacuccuccaacu..................   7     0   Fm1
........................................................................agauucaacuccuccaacugU................   5     1   Fm1
........................................................................agUuucaacuccuccaacugc................   1     1   Fm1
........................................................................agauucaacuccuccaCcugc................   1     1   Fm1
........................................................................aUauucaacuccuccaacugc................   1     1   Fm1
........................................................................Ugauucaacuccuccaacugc................   1     1   Fm1
........................................................................agauucaacuccuccaacugc................   136   0   Fm1
........................................................................agauucaacuUcuccaacugc................   1     1   Fm1
........................................................................agaAucaacuccuccaacugc................   1     1   Fm1
........................................................................agauAcaacuccuccaacugc................   1     1   Fm1
........................................................................agauucaacuccuccaaAugc................   1     1   Fm1
........................................................................agauucaacuccuccaacugA................   3     1   Fm1
........................................................................agauucaacuAcuccaacugcu...............   1     1   Fm1
........................................................................agauucaacuccuccaacugcu...............   29    0   Fm1
........................................................................agauucaacuccuccaaGugcu...............   1     1   Fm1
........................................................................agauucaacuccuccaaUugcu...............   1     1   Fm1
.........................................................................gauucaacuccuccaacu..................   15    0   Fm1
.........................................................................gauucaacuccuccaacG..................   1     1   Fm1
.........................................................................gauucaacuccuccaacugc................   4     0   Fm1
.........................................................................gauucaacuccuccaacugcu...............   4     0   Fm1
..........................................................................auucaacuccuccaacuU.................   1     1   Fm1
..........................................................................auucaacuccuccaacug.................   2     0   Fm1
..........................................................................auucaacuccuccaacugc................   9     0   Fm1
...........................................................................uucaacuccuccaacugc................   4     0   Fm1
...........................................................................uucaacuccuccaacugcG...............   1     1   Fm1
...........................................................................uucaacuccuccaacugcu...............   6     0   Fm1
............................................................................ucaacuccuccaacugcu...............   2     0   Fm1

.................................aguggagagaguuuuaAcucau......................................................   1     1   Fm2
.................................aguggagagaguuuuaucucau......................................................   9     0   Fm2
.................................aguggagagaguuuuauUucauc.....................................................   2     1   Fm2
.................................aguggagagaguuuuaucucauc.....................................................   99    0   Fm2
.................................Nguggagagaguuuuaucucauc.....................................................   1     1   Fm2
.................................aguggagagaAuuuuaucucauc.....................................................   1     1   Fm2
.....................................................................Cugagauucaacuccuccaacug.................   2     1   Fm2
.....................................................................Cugagauucaacuccuccaacugc................   4     1   Fm2
......................................................................ugagauucaacuccuccU.....................   6     1   Fm2
......................................................................ugagauucaacuccucca.....................   9     0   Fm2
......................................................................ugagauucaacuccuccaU....................   2     1   Fm2
......................................................................ugagauucaacuccuccaa....................   35    0   Fm2
......................................................................ugagauucaacuccuccCa....................   1     1   Fm2
......................................................................ugagauucaacuccucUaac...................   1     1   Fm2
......................................................................ugagauucaaGuccuccaac...................   1     1   Fm2
......................................................................ugagauucaacuccuccaac...................   63    0   Fm2
......................................................................ugagauucaacuccucAaac...................   1     1   Fm2
......................................................................ugagauucaacuccuccaaU...................   22    1   Fm2
......................................................................ugagauucaacuccucUaacu..................   4     1   Fm2
......................................................................ugagauucaacuccuccaacu..................   2028  0   Fm2
......................................................................ugagauucaacucAuccaacu..................   1     1   Fm2
......................................................................ugagauucaacuccuccGacu..................   7     1   Fm2
......................................................................ugagauucaacuccuccaacA..................   13    1   Fm2
......................................................................ugagauucaacuccucGaacu..................   1     1   Fm2
......................................................................ugagauucaacuUcuccaacu..................   3     1   Fm2
......................................................................ugagCuucaacuccuccaacu..................   2     1   Fm2
......................................................................ugagauucaacucUuccaacu..................   1     1   Fm2
......................................................................ugagauAcaacuccuccaacu..................   1     1   Fm2
......................................................................ugagauucaaGuccuccaacu..................   3     1   Fm2
......................................................................ugagauucaacuccucAaacu..................   10    1   Fm2
......................................................................ugagauuAaacuccuccaacu..................   1     1   Fm2
......................................................................ugagauucaaAuccuccaacu..................   2     1   Fm2
......................................................................ugagauucaacuccuccCacu..................   6     1   Fm2
......................................................................ugagauucaacuccuccaacC..................   6     1   Fm2
......................................................................Ggagauucaacuccuccaacu..................   1     1   Fm2
......................................................................ugagauucCacuccuccaacu..................   1     1   Fm2
......................................................................ugagauucaacuccuccaGcu..................   2     1   Fm2
......................................................................ugaAauucaacuccuccaacu..................   1     1   Fm2
......................................................................ugGgauucaacuccuccaacu..................   1     1   Fm2
......................................................................ugagauucaacuccuAcaacu..................   1     1   Fm2
......................................................................ugagUuucaacuccuccaacu..................   1     1   Fm2
......................................................................ugagauucaacuccuccaacG..................   55    1   Fm2
```

```
                                Star                                Mature
ggcuccuucccuucuuacugacacaucaagagaaguggagagaguuuuaucucaucgucugauauguuggugagauucaacuccuccaacugcucucggugugaaccau
......................................................................ugagauucaacAccuccaacu..................  1      1  Fm2
......................................................................ugagauucaacuccuccUacu..................  8      1  Fm2
......................................................................ugagauucaGcuccuccaacu..................  2      1  Fm2
......................................................................Ngagauucaacuccuccaacu..................  1      1  Fm2
......................................................................ugagauucaaUuccuccaacu..................  3      1  Fm2
......................................................................ugagUuucaacuccuccaacug.................  6      1  Fm2
......................................................................ugagauucaacucAuccaacug.................  5      1  Fm2
......................................................................ugagauucaacuccuccaacuA.................  8      1  Fm2
......................................................................ugagauucaacuccuAcaacug.................  1      1  Fm2
......................................................................ugagaNucaacuccuccaacug.................  3      1  Fm2
......................................................................ugagauucaacuUcuccaacug.................  2      1  Fm2
......................................................................ugagauucUacuccuccaacug.................  2      1  Fm2
......................................................................ugaCauucaacuccuccaacug.................  1      1  Fm2
......................................................................ugagauucaacuccuccaacug.................  9450   0  Fm2
......................................................................ugagauucaacuccuUcaacug.................  1      1  Fm2
......................................................................ugagauucaacuccuccaCcug.................  1      1  Fm2
......................................................................ugUgauucaacuccuccaacug.................  2      1  Fm2
......................................................................ugagauucaacuccuccUacug.................  30     1  Fm2
......................................................................ugagauucaGcuccuccaacug.................  1      1  Fm2
......................................................................ugagauucaaGuccuccaacug.................  18     1  Fm2
......................................................................ugagCuucaacuccuccaacug.................  20     1  Fm2
......................................................................ugagauucaacucGuccaacug.................  1      1  Fm2
......................................................................ugGgauucaacuccuccaacug.................  2      1  Fm2
......................................................................ugagauNcaacuccuccaacug.................  2      1  Fm2
......................................................................ugagGuucaacuccuccaacug.................  12     1  Fm2
......................................................................Cgagauucaacuccuccaacug.................  3      1  Fm2
......................................................................uAagauucaacuccuccaacug.................  7      1  Fm2
......................................................................ugagaAucaacuccuccaacug.................  1      1  Fm2
......................................................................ugagauucaUcuccuccaacug.................  1      1  Fm2
......................................................................ugagauucaacuccucGaacug.................  15     1  Fm2
......................................................................ugagauucaacuccucAaacug.................  49     1  Fm2
......................................................................ugagauucaaAuccuccaacug.................  16     1  Fm2
......................................................................uUagauucaacuccuccaacug.................  4      1  Fm2
......................................................................ugagauucGacuccuccaacug.................  3      1  Fm2
......................................................................ugagauucaacuccuccaacuC.................  1      1  Fm2
......................................................................Ngagauucaacuccuccaacug.................  5      1  Fm2
......................................................................ugagauGcaacuccuccaacug.................  1      1  Fm2
......................................................................ugagauucaacuccuccCacug.................  31     1  Fm2
......................................................................ugagauucaacuccuccaacuU.................  43     1  Fm2
......................................................................ugagauucaacuccuccaacAg.................  2      1  Fm2
......................................................................ugagaCucaacuccuccaacug.................  1      1  Fm2
......................................................................ugagauucaacuccuccGacug.................  32     1  Fm2
......................................................................ugagauucaacuGcuccaacug.................  1      1  Fm2
......................................................................ugagauucaacuccuccaUcug.................  1      1  Fm2
......................................................................ugagauucaacuccucUaacug.................  29     1  Fm2
......................................................................ugagauucaacuccCccaacug.................  1      1  Fm2
......................................................................ugagauucaacuccuccaacGg.................  170    1  Fm2
......................................................................ugagauucaacuAcuccaacug.................  1      1  Fm2
......................................................................ugagauucaacAccuccaacug.................  1      1  Fm2
......................................................................ugagauuAaacuccuccaacug.................  2      1  Fm2
......................................................................ugagauucaacuccuccaGcug.................  1      1  Fm2
......................................................................ugagauucaaUuccuccaacug.................  9      1  Fm2
......................................................................ugagauuUaacuccuccaacug.................  4      1  Fm2
......................................................................uCagauucaacuccuccaacug.................  1      1  Fm2
......................................................................ugagauucCacuccuccaacug.................  1      1  Fm2
......................................................................ugagauucaacuccuccaacGgc................  1      1  Fm2
......................................................................ugagauucUacuccuccaacugc................  36     1  Fm2
......................................................................ugagauucaacuccuccCacugc................  100    1  Fm2
......................................................................uAagauucaacuccuccaacugc................  18     1  Fm2
......................................................................ugagauucaacuccuccaacugU................  5592   1  Fm2
......................................................................ugagaGucaacuccuccaacugc................  3      1  Fm2
......................................................................ugagauuAaacuccuccaacugc................  6      1  Fm2
......................................................................ugNgauucaacuccuccaacugc................  1      1  Fm2
......................................................................ugagauucaacuccuccaUcugc................  6      1  Fm2
......................................................................ugagauucaacuccuccaGcugc................  3      1  Fm2
......................................................................ugagauucaacuccuccaacugc................  36097  0  Fm2
......................................................................ugagauucaacuccuccUacugc................  100    1  Fm2
......................................................................ugagauucaUcuccuccaacugc................  4      1  Fm2
......................................................................ugagauucaacuccuccGacugc................  125    1  Fm2
......................................................................ugGgauucaacuccuccaacugc................  9      1  Fm2
```

```
                                Star                                 Mature
ggcuccuucccuucuuacugacacaucaagagaaguggagagaguuuuaucucaucgucugauauguugguagagauucaacuccuccaacugcucucggugugaaccau
......................................................................ugagauucaacuccuAcaacugc................   5     1    Fm2
......................................................................ugagauucaaUuccuccaacugc................   49    1    Fm2
......................................................................ugagauucaacuGcuccaacugc................   5     1    Fm2
......................................................................uNagauucaacuccuccaacugc................   1     1    Fm2
......................................................................ugagauucaacucGuccaacugc................   8     1    Fm2
......................................................................ugagauAcaacuccuccaacugc................   4     1    Fm2
......................................................................ugagauuUaacuccuccaacugc................   10    1    Fm2
......................................................................ugagauucaacAccuccaacugc................   4     1    Fm2
......................................................................ugaAauucaacuccuccaacugc................   3     1    Fm2
......................................................................ugagauucaacuccuccaaUugc................   6     1    Fm2
......................................................................ugagauucaacuccuccaacuUc................   4     1    Fm2
......................................................................ugaCauucaacuccuccaacugc................   2     1    Fm2
......................................................................ugCgauucaacuccuccaacugc................   5     1    Fm2
......................................................................ugagauucaacuccuccaacAgc................   1     1    Fm2
......................................................................ugagaNucaacuccuccaacugc................   7     1    Fm2
......................................................................ugagauucaacuccuGcaacugc................   6     1    Fm2
......................................................................ugagauucaacuAcuccaacugc................   3     1    Fm2
......................................................................ugagaAucaacuccuccaacugc................   3     1    Fm2
......................................................................Cgagauucaacuccuccaacugc................   10    1    Fm2
......................................................................ugagauucaacuccGccaacugc................   1     1    Fm2
......................................................................ugagauNcaacuccuccaacugc................   4     1    Fm2
......................................................................uUagauucaacuccuccaacugc................   4     1    Fm2
......................................................................Ngagauucaacuccuccaacugc................   21    1    Fm2
......................................................................ugagauucaacCccuccaacugc................   4     1    Fm2
......................................................................ugagauucGacuccuccaacugc................   16    1    Fm2
......................................................................Agagauucaacuccuccaacugc................   2     1    Fm2
......................................................................ugagauucaacucUuccaacugc................   5     1    Fm2
......................................................................ugagauucaacuccuccaacugG................   157   1    Fm2
......................................................................ugagauucaacuccuccaacCgc................   5     1    Fm2
......................................................................ugagauucaaAuccuccaacugc................   46    1    Fm2
......................................................................ugagauucaaGuccuccaacugc................   37    1    Fm2
......................................................................ugaUauucaacuccuccaacugc................   3     1    Fm2
......................................................................ugagauucaCcuccuccaacugc................   4     1    Fm2
......................................................................ugagauucaacuccCccaacugc................   10    1    Fm2
......................................................................ugagauucaacuccuccaacuAc................   9     1    Fm2
......................................................................ugagauCcaacuccuccaacugc................   5     1    Fm2
......................................................................ugagauucaacuccucGaacugc................   44    1    Fm2
......................................................................ugagauuGaacuccuccaacugc................   3     1    Fm2
......................................................................ugagauGcaacuccuccaacugc................   1     1    Fm2
......................................................................ugagauucaGcuccuccaacugc................   6     1    Fm2
......................................................................ugagauucaacuccucAaacugc................   157   1    Fm2
......................................................................ugagauucaacuccuccaacugA................   39    1    Fm2
......................................................................ugagauucCacuccuccaacugc................   5     1    Fm2
......................................................................ugagauucaacuccuccaaGugc................   1     1    Fm2
......................................................................ugUgauucaacuccuccaacugc................   6     1    Fm2
......................................................................ugagCuucaacuccuccaacugc................   118   1    Fm2
......................................................................ugagGuucaacuccuccaacugc................   33    1    Fm2
......................................................................ugagaCucaacuccuccaacugc................   6     1    Fm2
......................................................................ugagauucaacGccuccaacugc................   1     1    Fm2
......................................................................uCagauucaacuccuccaacugc................   2     1    Fm2
......................................................................ugagauucaacuccuccaCcugc................   1     1    Fm2
......................................................................ugagauucaacuccucUaacugc................   106   1    Fm2
......................................................................ugagauucaacuUcuccaacugc................   4     1    Fm2
......................................................................ugagauucaacuccuUcaacugc................   1     1    Fm2
......................................................................ugagauucaacucAuccaacugc................   17    1    Fm2
......................................................................ugagUuucaacuccuccaacugc................   34    1    Fm2
......................................................................ugagauucaacuccAccaacugc................   1     1    Fm2
......................................................................ugagauucaaGuccuccaacugcu...............   3     1    Fm2
......................................................................ugagauucaacucAuccaacugcu...............   3     1    Fm2
......................................................................ugagauucaacuccuccaacugcA...............   335   1    Fm2
......................................................................ugagauucaacuccuccCacugcu...............   5     1    Fm2
......................................................................uCagauucaacuccuccaacugcu...............   1     1    Fm2
......................................................................ugagauucaacuccuccaacugcG...............   45    1    Fm2
......................................................................ugagauucaacuAcuccaacugcu...............   3     1    Fm2
......................................................................ugagauuUaacuccuccaacugcu...............   2     1    Fm2
......................................................................ugagauucaacuccucGaacugcu...............   7     1    Fm2
......................................................................ugagauucaacuccuccGacugcu...............   11    1    Fm2
......................................................................Cgagauucaacuccuccaacugcu...............   1     1    Fm2
......................................................................ugagauucaacuccucAaacugcu...............   9     1    Fm2
......................................................................ugagauucaacuccuccaacuAcu...............   1     1    Fm2
```

Star   Mature

```
ggcuccuucccuucuuacugacacaucaagagaaguggagagaguuuuaucucaucgucugauauguuggugagauucaacuccuccaacugcucucggugugaaccau
......................................................................ugagauucGacuccuccaacugcu...............   1      1   Fm2
......................................................................ugagauucaaAuccuccaacugcu...............   4      1   Fm2
......................................................................ugagauucaacuccuccaacugcu...............   2154   0   Fm2
......................................................................ugagauucaacGccuccaacugcu...............   1      1   Fm2
......................................................................ugagauucaacucGuccaacugcu...............   2      1   Fm2
......................................................................ugagauucaUcuccuccaacugcu...............   1      1   Fm2
......................................................................ugagaCucaacuccuccaacugcu...............   1      1   Fm2
......................................................................uUagauucaacuccuccaacugcu...............   1      1   Fm2
......................................................................ugagauucaacuccuccaacugcC...............   1      1   Fm2
......................................................................ugagUuucaacuccuccaacugcu...............   1      1   Fm2
......................................................................ugagauucaacuccucUaacugcu...............   23     1   Fm2
......................................................................ugagauucUacuccuccaacugcu...............   2      1   Fm2
......................................................................ugagauucaacuccuGcaacugcu...............   1      1   Fm2
......................................................................ugagauucaacuccuccUacugcu...............   8      1   Fm2
......................................................................ugagauucaacuccuccaacCgcu...............   1      1   Fm2
......................................................................ugagauucaacuccuccaUcugcu...............   1      1   Fm2
......................................................................ugagauucaacuccuccaacugUu...............   311    1   Fm2
.......................................................................gagauucaacuccuccaacu..................   1      0   Fm2
.......................................................................gagauucaacuccuccaacug.................   5      0   Fm2
.......................................................................gagauucaacuccuccaacugc................   3      0   Fm2
.......................................................................gagauucaacuccuccaacugcu...............   4      0   Fm2
........................................................................agauucaacuccuccaacu..................   1      0   Fm2
........................................................................agGuucaacuccuccaacugc................   2      1   Fm2
........................................................................agauucaacuccuccaaUugc................   1      1   Fm2
........................................................................agauucaacuccuccaacugc................   43     0   Fm2
........................................................................agCuucaacuccuccaacugc................   1      1   Fm2
........................................................................agauucaacuccuccaaAugc................   1      1   Fm2
........................................................................Ugauucaacuccuccaacugc................   1      1   Fm2
........................................................................agauucaacuccuccaacugU................   2      1   Fm2
.........................................................................gauucaacuccuccaacugU................   1      1   Fm2
...........................................................................uucaacuccuccaacugc................   1      0   Fm2

.................................aguggagagagCuuuaucucau......................................................   1      1   Mm2
.................................aguggagagaguuuuaucucauc.....................................................   3      0   Mm2
......................................................................ugagauucaacuccuccaac...................   9      0   Mm2
......................................................................ugagauucaaUuccuccaacu..................   1      1   Mm2
......................................................................ugagauucaacuccuccGacu..................   1      1   Mm2
......................................................................ugagauucaacuccuccaacG..................   7      1   Mm2
......................................................................ugagauucaacuccuccUacu..................   1      1   Mm2
......................................................................ugagauucaacuccuccaacA..................   1      1   Mm2
......................................................................ugagauucaacuccuccaaUu..................   1      1   Mm2
......................................................................ugagauucaacuccuccCacu..................   2      1   Mm2
......................................................................ugagauucaacuccuccaacu..................   147    0   Mm2
......................................................................Cgagauucaacuccuccaacug.................   1      1   Mm2
......................................................................ugagauucaacuccucAaacug.................   3      1   Mm2
......................................................................ugagauucaacuccuGcaacug.................   1      1   Mm2
......................................................................ugagauucaacuUcuccaacug.................   1      1   Mm2
......................................................................ugagauucaaGuccuccaacug.................   1      1   Mm2
......................................................................Ngagauucaacuccuccaacug.................   2      1   Mm2
......................................................................ugagauucaacuccuccaacGg.................   15     1   Mm2
......................................................................ugagauucaacuccucGaacug.................   1      1   Mm2
......................................................................ugagauucaacuccuccCacug.................   2      1   Mm2
......................................................................ugagauucaacuccuccaacug.................   481    0   Mm2
......................................................................ugagauucaacuccucUaacug.................   1      1   Mm2
......................................................................uUagauucaacuccuccaacug.................   1      1   Mm2
......................................................................ugagauucaacuccuccaacuU.................   3      1   Mm2
......................................................................ugagauucaacuccuccaacuA.................   2      1   Mm2
......................................................................ugagauucaacuccuccGacug.................   2      1   Mm2
......................................................................ugagauucaacuccuccUacug.................   1      1   Mm2
......................................................................ugagauucaaUuccuccaacugc................   3      1   Mm2
......................................................................ugagaAucaacuccuccaacugc................   1      1   Mm2
......................................................................ugagauucaacuccuccaacCgc................   1      1   Mm2
......................................................................ugagauucaacuccuUcaacugc................   1      1   Mm2
......................................................................ugagauuUaacuccuccaacugc................   1      1   Mm2
......................................................................ugaCauucaacuccuccaacugc................   1      1   Mm2
......................................................................ugagauucaacuccuccCacugc................   3      1   Mm2
......................................................................ugagauucaacuccuccaacugc................   1857   0   Mm2
......................................................................ugagauucaacuccuccaacugU................   410    1   Mm2
......................................................................ugagauucaacuccucAaacugc................   10     1   Mm2
......................................................................uAagauucaacuccuccaacugc................   2      1   Mm2
```

```
                                 Star                                Mature
ggcuccuucccuucuuacugacacaucaagagaaguggagagaguuuuaucucaucgucugauauguuggugagauucaacuccuccaacugcucucggugugaaccau
......................................................................ugagauucaaAuccuccaacugc................   2     1    Mm2
......................................................................ugagauucaacuccuccaacugG................   5     1    Mm2
......................................................................ugagauucaacuGcuccaacugc................   1     1    Mm2
......................................................................ugagauucUacuccuccaacugc................   1     1    Mm2
......................................................................ugagauucaacuccucGaacugc................   1     1    Mm2
......................................................................ugGgauucaacuccuccaacugc................   2     1    Mm2
......................................................................ugagauucaacuccucUaacugc................   8     1    Mm2
......................................................................ugagauAcaacuccuccaacugc................   1     1    Mm2
......................................................................ugagauucaacuccuccGacugc................   8     1    Mm2
......................................................................ugagauucaacucAuccaacugc................   1     1    Mm2
......................................................................ugagauucaacuccuccUacugc................   4     1    Mm2
......................................................................ugagauucaaGuccuccaacugc................   1     1    Mm2
......................................................................ugagauucaacuccuccaacugA................   4     1    Mm2
......................................................................ugagauucaacCccuccaacugc................   1     1    Mm2
......................................................................ugagauucGacuccuccaacugc................   1     1    Mm2
......................................................................ugagauucaacuccucAaacugcu...............   1     1    Mm2
......................................................................ugagauucaacuccuccCacugcu...............   2     1    Mm2
......................................................................ugagauucaacuccuccaacugUu...............   13    1    Mm2
......................................................................ugagauucaacuccuccaacugcG...............   6     1    Mm2
......................................................................ugagauucaacuccuccaacugcA...............   16    1    Mm2
......................................................................ugagauucaacuccucUaacugcu...............   3     1    Mm2
......................................................................ugagauucaacuccuccaacugcu...............   160   0    Mm2
.......................................................................gagauucaacuccuccaacugU................   1     1    Mm2
.......................................................................gagauucaacuccuccaacugc................   1     0    Mm2
........................................................................agauucaacuccuccaacugU................   2     1    Mm2
........................................................................agauucaacuccuccaacugc................   7     0    Mm2
........................................................................agauucaacuccuccaacugcA...............   1     1    Mm2
........................................................................agauucaacuccuccaacugcC...............   1     1    Mm2

.................................aguggagagaguuuuaucucau......................................................   2     0    Mm1
.................................aguggagagaCuuuuaucucauc.....................................................   1     1    Mm1
.................................aguggagagaguuuuaucucauc.....................................................   15    0    Mm1
.....................................................................Cugagauucaacuccuccaacu..................   3     1    Mm1
.....................................................................Cugagauucaacuccuccaacug.................   2     1    Mm1
.....................................................................Cugagauucaacuccuccaacugc................   5     1    Mm1
.....................................................................Cugagauucaacuccuccaacugcu...............   2     1    Mm1
......................................................................ugagauucaacuccucAa.....................   1     1    Mm1
......................................................................ugagauucaacuccucca.....................   21    0    Mm1
......................................................................ugagauucaaAuccuccaa....................   1     1    Mm1
......................................................................ugagauucaacuccuccaU....................   9     1    Mm1
......................................................................ugagauucaacuccuccaa....................   27    0    Mm1
......................................................................ugagauucaacuccuccaaA...................   1     1    Mm1
......................................................................ugagauucaacuccucUaac...................   2     1    Mm1
......................................................................ugagauucaacuccuccGac...................   1     1    Mm1
......................................................................ugagauucaacuccuccaaU...................   6     1    Mm1
......................................................................ugagauucaacuccuccaac...................   141   0    Mm1
......................................................................ugagauucaacuccuUcaacu..................   1     1    Mm1
......................................................................ugagauuUaacuccuccaacu..................   3     1    Mm1
......................................................................ugagauucaacuccucUaacu..................   16    1    Mm1
......................................................................ugagauucaacuccuccGacu..................   25    1    Mm1
......................................................................uUagauucaacuccuccaacu..................   1     1    Mm1
......................................................................ugagauucaacuccucGaacu..................   7     1    Mm1
......................................................................ugagauucaCcuccuccaacu..................   1     1    Mm1
......................................................................ugagauucaacuUcuccaacu..................   2     1    Mm1
......................................................................ugagauucaacuccCccaacu..................   1     1    Mm1
......................................................................ugagaCucaacuccuccaacu..................   1     1    Mm1
......................................................................ugagauucaacuccuccaGcu..................   1     1    Mm1
......................................................................ugagGuucaacuccuccaacu..................   2     1    Mm1
......................................................................ugagauucaacuccuccCacu..................   13    1    Mm1
......................................................................ugagauucaacuccuccaacC..................   2     1    Mm1
......................................................................ugagauucaaAuccuccaacu..................   7     1    Mm1
......................................................................ugagauucaacuccuccaacA..................   32    1    Mm1
......................................................................ugagauucUacuccuccaacu..................   2     1    Mm1
......................................................................ugagauucaaUuccuccaacu..................   2     1    Mm1
......................................................................ugagauucaacuccuccaacu..................   4927  0    Mm1
......................................................................ugagauCcaacuccuccaacu..................   1     1    Mm1
......................................................................ugagCuucaacuccuccaacu..................   3     1    Mm1
......................................................................ugagauucaacucAuccaacu..................   2     1    Mm1
......................................................................ugagauucaaGuccuccaacu..................   4     1    Mm1
......................................................................ugagUuucaacuccuccaacu..................   3     1    Mm1
```

Star Mature

```
ggcuccuucccuucuuacugacacaucaagagaaguggagagaguuuuaucucaucgucugauauguuggugagauucaacuccuccaacugcucucggugugaaccau
......................................................................ugagauucaacuccucAaacu...................   35      1      Mm1
......................................................................ugagauucaacuGcuccaacu...................   1       1      Mm1
......................................................................ugagauucaacuccuccUacu...................   10      1      Mm1
......................................................................ugagauucaGcuccuccaacu...................   1       1      Mm1
......................................................................Agagauucaacuccuccaacu...................   1       1      Mm1
......................................................................ugaUauucaacuccuccaacu...................   1       1      Mm1
......................................................................ugagauucaacucUuccaacu...................   2       1      Mm1
......................................................................ugGgauucaacuccuccaacu...................   3       1      Mm1
......................................................................ugagauucCacuccuccaacu...................   1       1      Mm1
......................................................................uCagauucaacuccuccaacu...................   1       1      Mm1
......................................................................ugagauucaacuccuccaacG...................   10      1      Mm1
......................................................................ugagauucaacGccuccaacug..................   1       1      Mm1
......................................................................ugagauucaCcuccuccaacug..................   2       1      Mm1
......................................................................ugagUuucaacuccuccaacug..................   3       1      Mm1
......................................................................ugagauucaacuccuccaacuA..................   21      1      Mm1
......................................................................ugGgauucaacuccuccaacug..................   8       1      Mm1
......................................................................ugagauucaacuccuccaacCg..................   1       1      Mm1
......................................................................ugagauucaacuAcuccaacug..................   2       1      Mm1
......................................................................ugagauucaacuccuccUacug..................   66      1      Mm1
......................................................................ugagauucaacuUcuccaacug..................   4       1      Mm1
......................................................................ugagauucGacuccuccaacug..................   8       1      Mm1
......................................................................uUagauucaacuccuccaacug..................   1       1      Mm1
......................................................................ugagauucaacuccCccaacug..................   2       1      Mm1
......................................................................ugagauucaacuccucAaacug..................   98      1      Mm1
......................................................................ugagCuucaacuccuccaacug..................   6       1      Mm1
......................................................................ugagauucCacuccuccaacug..................   2       1      Mm1
......................................................................ugagaCucaacuccuccaacug..................   2       1      Mm1
......................................................................ugagaNucaacuccuccaacug..................   3       1      Mm1
......................................................................ugagauucaacCccuccaacug..................   9       1      Mm1
......................................................................ugagauucaacuGcuccaacug..................   1       1      Mm1
......................................................................ugagaAucaacuccuccaacug..................   2       1      Mm1
......................................................................ugaAauucaacuccuccaacug..................   2       1      Mm1
......................................................................ugagauNcaacuccuccaacug..................   3       1      Mm1
......................................................................ugaUauucaacuccuccaacug..................   1       1      Mm1
......................................................................ugagauucaacucUuccaacug..................   6       1      Mm1
......................................................................ugagauucaUcuccuccaacug..................   2       1      Mm1
......................................................................ugagauucaacuccucUaacug..................   76      1      Mm1
......................................................................ugagauucaaAuccuccaacug..................   28      1      Mm1
......................................................................ugagauucaaGuccuccaacug..................   33      1      Mm1
......................................................................ugagauucaGcuccuccaacug..................   3       1      Mm1
......................................................................Cgagauucaacuccuccaacug..................   1       1      Mm1
......................................................................ugagauGcaacuccuccaacug..................   1       1      Mm1
......................................................................ugagauucUacuccuccaacug..................   8       1      Mm1
......................................................................ugagauucaacuccuAcaacug..................   2       1      Mm1
......................................................................ugagauucaacuccuccaacuU..................   43      1      Mm1
......................................................................Ngagauucaacuccuccaacug..................   7       1      Mm1
......................................................................ugagauucaacuccuccaaUug..................   1       1      Mm1
......................................................................ugagauucaacuccuccGacug..................   68      1      Mm1
......................................................................ugagauucaacuccuGcaacug..................   2       1      Mm1
......................................................................ugagauucaacuccucGaacug..................   24      1      Mm1
......................................................................ugagauAcaacuccuccaacug..................   1       1      Mm1
......................................................................ugagaGucaacuccuccaacug..................   1       1      Mm1
......................................................................ugagauuUaacuccuccaacug..................   13      1      Mm1
......................................................................ugagauucaacuccuccCacug..................   53      1      Mm1
......................................................................ugagauucaacuccuccaGcug..................   2       1      Mm1
......................................................................ugagGuucaacuccuccaacug..................   8       1      Mm1
......................................................................ugUgauucaacuccuccaacug..................   3       1      Mm1
......................................................................ugagauCcaacuccuccaacug..................   2       1      Mm1
......................................................................ugagauucaaUuccuccaacug..................   26      1      Mm1
......................................................................ugagauucaacuccuccaacug..................   20089   0      Mm1
......................................................................ugagauucaacucAuccaacug..................   4       1      Mm1
......................................................................ugagauucaacuccuccaacGg..................   22      1      Mm1
......................................................................uAagauucaacuccuccaacug..................   4       1      Mm1
......................................................................ugagauucaacuccuccaUcug..................   1       1      Mm1
......................................................................ugagauucaacuccuUcaacug..................   6       1      Mm1
......................................................................uAagauucaacuccuccaacugc.................   35      1      Mm1
......................................................................ugagauucaacuccuccaacuCc.................   1       1      Mm1
......................................................................ugagauucaacuccuccaCcugc.................   2       1      Mm1
......................................................................ugagauucaacuccuccaaAugc.................   1       1      Mm1
......................................................................Ngagauucaacuccuccaacugc.................   32      1      Mm1
```

```
                                Star                                Mature
ggcuccuucccuucuuacugacacaucaagagaaguggagagaguuuuaucucaucgucugauauguuggugagauucaacuccuccaacugcucucggugugaaccau
......................................................................ugagauucaaUuccuccaacugc................ 62     1 Mm1
......................................................................ugagauuGaacuccuccaacugc................ 5      1 Mm1
......................................................................ugagauucaacuccucAaacugc................ 335    1 Mm1
......................................................................ugagauNcaacuccuccaacugc................ 6      1 Mm1
......................................................................ugagauucaCcuccuccaacugc................ 7      1 Mm1
......................................................................ugagaAucaacuccuccaacugc................ 5      1 Mm1
......................................................................ugagauucaacuccuccaacCgc................ 7      1 Mm1
......................................................................ugaUauucaacuccuccaacugc................ 4      1 Mm1
......................................................................ugGgauucaacuccuccaacugc................ 23     1 Mm1
......................................................................ugUgauucaacuccuccaacugc................ 9      1 Mm1
......................................................................ugagauucaacuccuccCacugc................ 193    1 Mm1
......................................................................ugagUuucaacuccuccaacugc................ 9      1 Mm1
......................................................................ugagauucCacuccuccaacugc................ 4      1 Mm1
......................................................................ugCgauucaacuccuccaacugc................ 5      1 Mm1
......................................................................ugagauCcaacuccuccaacugc................ 9      1 Mm1
......................................................................Ggagauucaacuccuccaacugc................ 1      1 Mm1
......................................................................ugagauuUaacuccuccaacugc................ 32     1 Mm1
......................................................................ugagauucaacuAcuccaacugc................ 11     1 Mm1
......................................................................ugagauucaacuccuGcaacugc................ 1      1 Mm1
......................................................................ugaCauucaacuccuccaacugc................ 1      1 Mm1
......................................................................ugagauucaacuccuccaacuAc................ 7      1 Mm1
......................................................................ugagauucaacuccucUaacugc................ 211    1 Mm1
......................................................................ugagauucaacuUcuccaacugc................ 19     1 Mm1
......................................................................ugagauucaacuccuccaacugc................ 70407  0 Mm1
......................................................................ugagauucaacuccuccaacAgc................ 2      1 Mm1
......................................................................ugagauucaacuccuccaaUugc................ 9      1 Mm1
......................................................................ugagaNucaacuccuccaacugc................ 12     1 Mm1
......................................................................ugagauucaacuccuccaacugU................ 10476  1 Mm1
......................................................................ugagauucaacucUuccaacugc................ 8      1 Mm1
......................................................................ugagauucaacucAuccaacugc................ 14     1 Mm1
......................................................................ugagauucaacuccucGaacugc................ 112    1 Mm1
......................................................................uNagauucaacuccuccaacugc................ 1      1 Mm1
......................................................................ugagauucaaAuccuccaacugc................ 119    1 Mm1
......................................................................ugagauucaGcuccuccaacugc................ 11     1 Mm1
......................................................................ugagauucGacuccuccaacugc................ 21     1 Mm1
......................................................................ugagauuAaacuccuccaacugc................ 7      1 Mm1
......................................................................ugagauucaacuccuccaacugA................ 133    1 Mm1
......................................................................ugNgauucaacuccuccaacugc................ 1      1 Mm1
......................................................................ugagGuucaacuccuccaacugc................ 16     1 Mm1
......................................................................ugagauucaacuccCccaacugc................ 4      1 Mm1
......................................................................ugagauucaacuccuccaacuUc................ 4      1 Mm1
......................................................................ugagauucaacuccuGcaacugc................ 270    1 Mm1
......................................................................ugagauucaacuccuccaacugG................ 38     1 Mm1
......................................................................uUagauucaacuccuccaacugc................ 16     1 Mm1
......................................................................ugagauucaacucGuccaacugc................ 5      1 Mm1
......................................................................ugagaCucaacuccuccaacugc................ 15     1 Mm1
......................................................................ugagauucaacuccuAcaacugc................ 11     1 Mm1
......................................................................ugaAauucaacuccuccaacugc................ 9      1 Mm1
......................................................................ugagauucaUcuccuccaacugc................ 8      1 Mm1
......................................................................ugagauucaacuccucUacugc................ 240    1 Mm1
......................................................................ugagauucaacGccuccaacugc................ 1      1 Mm1
......................................................................Cgagauucaacuccuccaacugc................ 6      1 Mm1
......................................................................ugagauucaacCccuccaacugc................ 7      1 Mm1
......................................................................ugagauucaacuccuccaUcugc................ 11     1 Mm1
......................................................................ugagauucaacAccuccaacugc................ 8      1 Mm1
......................................................................uCagauucaacuccuccaacugc................ 6      1 Mm1
......................................................................ugagauucaacuGcuccaacugc................ 4      1 Mm1
......................................................................ugagauucaacuccAccaacugc................ 4      1 Mm1
......................................................................ugagauGcaacuccuccaacugc................ 1      1 Mm1
......................................................................ugagauucaaGuccuccaacugc................ 98     1 Mm1
......................................................................ugagauAcaacuccuccaacugc................ 7      1 Mm1
......................................................................Agagauucaacuccuccaacugc................ 7      1 Mm1
......................................................................ugagaGucaacuccuccaacugc................ 1      1 Mm1
......................................................................ugagauucaacuccuUcaacugc................ 14     1 Mm1
......................................................................ugagCuucaacuccuccaacugc................ 55     1 Mm1
......................................................................ugagauucaacuccuccaGcugc................ 13     1 Mm1
......................................................................ugagauucUacuccuccaacugc................ 66     1 Mm1
......................................................................ugagauucaacuccuccaacGgc................ 1      1 Mm1
......................................................................ugagauucaaGuccuccaacugcu............... 2      1 Mm1
......................................................................ugagauucaacuccuccaacugAu............... 3      1 Mm1
```

```
                                  Star                                Mature
ggcuccuucccuucuuacugacacaucaagagaaguggagagaguuuuaucucaucgucugauauguuggugagauucaacuccuccaacugcucucggugugaaccau
......................................................................Cgagauucaacuccuccaacugcu...............   1      1    Mm1
......................................................................ugagauucaacuccuAcaacugcu...............   1      1    Mm1
......................................................................ugagauucaacucUuccaacugcu...............   1      1    Mm1
......................................................................ugagauucaacuccuUcaacugcu...............   1      1    Mm1
......................................................................uAagauucaacuccuccaacugcu...............   2      1    Mm1
......................................................................ugagGuucaacuccuccaacugcu...............   1      1    Mm1
......................................................................ugagauucaacuccuccaacGgcu...............   1      1    Mm1
......................................................................ugagauucaacuccuccaacAgcu...............   1      1    Mm1
......................................................................ugagaAucaacuccuccaacugcu...............   1      1    Mm1
......................................................................ugagauucaacuccuccGacugcu...............   14     1    Mm1
......................................................................ugagaGucaacuccuccaacugcu...............   1      1    Mm1
......................................................................ugagauucaacuccCccaacugcu...............   1      1    Mm1
......................................................................ugagauucaaUuccuccaacugcu...............   8      1    Mm1
......................................................................ugagaNucaacuccuccaacugcu...............   1      1    Mm1
......................................................................ugagauucaacuccuccaacugcu...............   3899   0    Mm1
......................................................................ugagauucaacuccucGaacugcu...............   8      1    Mm1
......................................................................ugagauucaacAccuccaacugcu...............   1      1    Mm1
......................................................................ugagaCucaacuccuccaacugcu...............   1      1    Mm1
......................................................................ugagauucaacuccuccUacugcu...............   6      1    Mm1
......................................................................ugagauucaacuccuccaacugUu...............   446    1    Mm1
......................................................................ugaUauucaacuccuccaacugcu...............   1      1    Mm1
......................................................................ugagauucGacuccuccaacugcu...............   2      1    Mm1
......................................................................ugagauuUaacuccuccaacugcu...............   1      1    Mm1
......................................................................ugagauucaaAuccuccaacugcu...............   4      1    Mm1
......................................................................ugagauucaacuccucUaacugcu...............   22     1    Mm1
......................................................................ugagauucaacucAuccaacugcu...............   2      1    Mm1
......................................................................ugagauucaacuccucAaacugcu...............   17     1    Mm1
......................................................................ugagauucaacuccuccaacugcC...............   19     1    Mm1
......................................................................ugagauucUacuccuccaacugcu...............   1      1    Mm1
......................................................................uUagauucaacuccuccaacugcu...............   1      1    Mm1
......................................................................Ngagauucaacuccuccaacugcu...............   2      1    Mm1
......................................................................ugagauucaacuccuccaacugcG...............   26     1    Mm1
......................................................................ugagauucaacuccuccaacugcA...............   845    1    Mm1
......................................................................ugagauucaacuccuccCacugcu...............   19     1    Mm1
......................................................................ugagauucaacuccuccaacugcuG..............   1      1    Mm1
......................................................................ugagauucaacuccuccaacugcuA..............   6      1    Mm1
.......................................................................gagauucaacuccuccaacu..................   5      0    Mm1
.......................................................................gagauucaacuccuccaacug.................   11     0    Mm1
.......................................................................Uagauucaacuccuccaacugc................   1      1    Mm1
.......................................................................gagauucaacuccuccaacugU................   7      1    Mm1
.......................................................................gagGuucaacuccuccaacugc................   1      1    Mm1
.......................................................................gagauucaacuccuccaacugc................   10     0    Mm1
.......................................................................gagauucaacuccuccaacugcu...............   4      0    Mm1
.......................................................................gagauucaacuccuccaacugUu...............   1      1    Mm1
........................................................................agauucaacuccuccaGcugc................   1      1    Mm1
........................................................................Ugauucaacuccuccaacugc................   1      1    Mm1
........................................................................agauucaacuccuccaacugc................   51     0    Mm1
........................................................................agauucaacuccuccaacugU................   2      1    Mm1
.........................................................................gauucaacuccuccaacu..................   4      0    Mm1
.........................................................................gauucaacuccuccaacug.................   10     0    Mm1
.........................................................................gauucaacuccuccaacugU................   4      1    Mm1
..........................................................................auucaacuccuccaacugU................   2      1    Mm1
...........................................................................uucaacuccuccaacugG................   1      1    Mm1
...........................................................................uucaacuccuccaacugU................   6      1    Mm1
...........................................................................uucaacuccuccaacugc................   9      0    Mm1
```
